# Care management intervention to strengthen self-care of multimorbid patients with type 2 diabetes in a German primary care network: A randomized controlled trial

**DOI:** 10.1371/journal.pone.0214056

**Published:** 2019-06-12

**Authors:** Dominik Ose, Martina Kamradt, Marion Kiel, Tobias Freund, Werner Besier, Manfred Mayer, Johannes Krisam, Michel Wensing, Hans-Joachim Salize, Joachim Szecsenyi

**Affiliations:** 1 University of Utah, Department of Family and Preventive Medicine, Salt Lake City, UT, United States of America; 2 University Hospital Heidelberg; Department of General Practice and Health Services Research; Marsilius-Arkaden, Turm West, Heidelberg, Germany; 3 Genossenschaft Gesundheitsprojekt Mannheim e.G., Mannheim, Germany; 4 University Hospital Heidelberg, Institute of Medical Biometry and Informatics, Marsilius-Arkaden, Turm West, Heidelberg, Germany; 5 Central Institute of Mental Health, Medical Faculty Mannheim / Heidelberg University, Mannheim, Germany; Florida International University Herbert Wertheim College of Medicine, UNITED STATES

## Abstract

**Purpose:**

This study aimed to assess the effectiveness of a care management intervention in improving self-management behavior in multimorbid patients with type 2 diabetes; care was delivered by medical assistants in the context of a primary care network (PCN) in Germany.

**Methods:**

This study is an 18-month, multi-center, two-armed, open-label, patient-randomized parallel-group superiority trial (ISRCTN 83908315). The intervention group received the care management intervention in addition to the usual care. The control group received usual care only. The primary outcome was the change in self-care behavior at month 9 compared to baseline. The self-care behavior was measured with the German version of the Summary of Diabetes Self-Care Activities Measure (SDSCA-G). A multilevel regression analysis was applied.

**Results:**

We assigned 495 patients to intervention (*n* = 252) and control (*n* = 243). At baseline, the mean age was 68 ±11 years, 47.8% of the patients were female and the mean HbA1c was 7.1±1.2%. The primary analysis showed no statistically significant effect, but a positive trend was observed (p = 0.206; 95%-CI = -0.084; 0.384). The descriptive analysis revealed a significantly increased sum score of the SDSCA-G in the intervention group over time (P = 0.012) but not in the control group (p = 0.1973).

**Conclusion:**

The sum score for self-care behavior markedly improved in the intervention group over time. However, the results of our primary analysis showed no statistically significant effect.

Possible reasons are the high baseline performance in our sample and the low intervention fidelity. The implementation of this care management intervention in PCNs has the potential to improve self-care behavior of multimorbid patients with type 2 diabetes.

## Introduction

Chronic illness care often includes day-to-day self-care responsibilities for patients [1]. This is especially true for diabetes. Diabetes self-care comprises a broad range of tasks such as self-monitoring of blood glucose, being physically active or controlling one’s feet on a regular basis [2]. Collaborative relationships with health care providers can help patients in handling and managing these self-care tasks [3]. Primary care can play a crucial role in supporting patients’ self-care as part of comprehensive chronic care management [4].

In Germany, a primary care-based disease management program (DMP) for type 2 diabetes was rolled out nationwide in 2002 [5]. This program comprises (among other elements) evidence-based clinical guidelines and quarterly visits to a primary care provider, as well as eye and foot exams on a regular basis [5, 6]. The German DMP for type 2 diabetes is free for patients with statutory or private health insurance. By 2015, about 4 million patients with type 2 diabetes were enrolled [7]. It is estimated that 5.8 million people with type 2 diabetes live in Germany [8].

However, dealing with co-morbidity in this German DMP is an enormous challenge, especially for those patients with type 2 diabetes, and nearly 90% of enrolled patients suffer from one or more co-occurring medical condition [9]. Co-morbidities are not unique to Germany, and primary care often has to face difficult challenges in caring for patients with multiple chronic conditions [10, 11]. There is an increasing prevalence of multiple, co-occurring conditions, especially for patients with severe diseases like diabetes mellitus [12, 13], which strongly influences the delivery of care [14, 15]. Comorbidity is demanding for both healthcare systems and patients; it demands complex clinical management and increasing health care costs [16–18], as well as impaired health-related quality of life (HRQoL).

Based on concepts for the re-organization of chronic care [19, 20], care management interventions have been developed that focus on patients with multiple chronic conditions. Care management has been defined as a set of interventions (e.g., comprehensive assessment of patients’ medical and nonmedical needs, monitoring of individualized, evidence-based care plans) [21] designed to assist patients in managing medical conditions and related psychosocial problems more effectively [22–24].

In Germany, the implementation of these concepts in office-based primary care practices (PCP) has been evaluated with promising results for patients with osteoarthritis [25], depression [26], chronic heart failure [27], and multi-morbidities [28]. Nevertheless, in small primary care settings (solo practices or 2-person partnerships) resources are often limited and extensive collaborative models, like care management, may be difficult to implement [28].

Primary care network (PCN) based approaches might ameliorate these challenges. PCNs can facilitate the sharing of resources and reduce the organizational workload of practices [29]. In Germany, PCNs are a newer model of primary care that focuses on improved access to care and the use of multidisciplinary teams for patients with chronic disease. PCNs consists of primary care physicians (PCP) and other providers working together to improve patient care [30]. In Germany, the number of PCNs has doubled from 200 to 400 in the last decade. The result is 30,000 physicians in PCNs, which in turn provides a strong foundation for population-based approaches [31, 32]. PCNs have also been successfully implemented in Canada and the US in recent years [33–35].

The primary objective in this study is to assess the effectiveness of a PCN based, IT-supported care management intervention with integrated telephone monitoring for the improvement of self-care behavior among patients with type 2 diabetes mellitus and multimorbidity. We hypothesize that this intervention will strengthen the self-care of multimorbid patients with type 2 diabetes. We chose patients with multimorbidities because the potential for improvements could be higher and the impact of structured care may be stronger compared to patients without multimorbidities.

## Research design and methods

This study (01/02/2014 to 31/07/2015) was designed as an 18-month, multi-center, two-armed, open-label, parallel-group superiority, randomized controlled trial (RCT). All participants were enrolled in the German DMP for patients with type 2 diabetes. The study office was located at the Department of General Practice and Health Services Research Heidelberg, which was responsible for coordination, data management, randomization, and monitoring. The intervention coordinating office was located at the ‘Genossenschaft Gesundheitsprojekt Mannheim’ (GGM) and was responsible for data entry, validation, and administration. The study was approved by the ethics committee of the Medical Faculty, Heidelberg University (S-590/2013). The research protocol has been published elsewhere [36]. No changes to methods or outcomes were made after trial commencement.

### Study center

The PCN is one of three in Mannheim, a city with approximately 305,000 inhabitants in the southwestern part of Germany. For participation in this study and to serve as a study center, PCN physicians had to meet the following criteria: (1) Specialized in general practice, internal medicine, or practical physician (2) working as a primary care physician according to German regulations and (3) being part of the PCP network GGM. Participating practices remained clinically and financially independent, but shared a number of facilities. Both single-handed and group practices were eligible to participate. PCPs who did not fulfill the inclusion criteria were excluded. All PCPs within the network were invited by the GGM management via an official letter to participate in the study. All PCPs in this study gave their written consent for participation.

### Participants

Patients older than 18-years old who met all of the following inclusion criteria were eligible to participate in the study: (1) officially diagnosed with type 2 diabetes mellitus (ICD 10: E11-E14), (2) enrolled in the DMP Diabetes mellitus type 2 (DMP Diabetes), and (3) diagnosed with at least two other severe chronic comorbidities according to the definition outlined in § 62 SGB V. These chronic comorbidities include, but are not limited to, atherosclerosis (ICD 10: I70), chronic coronary heart disease (ICD 10: I25), chronic obstructive lung disease (ICD 10: J44), asthma (ICD 10: J45), cerebrovascular diseases (ICD 10: I60-I69), depression (ICD 10: F32-F33), heart failure (ICD 10: I50), Parkinson’s (ICD 10: G20), and/or chronic pain (ICD 10: R52). Additionally, written informed consent was a prerequisite for participation in the study.

Patients who did not fulfill the inclusion criteria were excluded. In addition, the following exclusion criteria for patients were applied: Severe acute psychiatric disorders, e.g., schizophrenia, schizotypal and delusional disorders (ICD 10:F20-F29); dementia (ICD 10:F00-F03); mental and behavioral disorders due to psychoactive substance use (ICD 10:F11-F16; F18; F19); malignant neoplasms (ICD 10:C00-C97), and/or current chemotherapy or radiotherapy; transplanted organ and tissue status (ICD 10:Z94); care involving dialysis (ICD 10:Z49); insurmountable language or communication problems; emergency cases.

### Recruitment

The recruitment of patients took place between 01/02/2014 and 31/10/2014 (T0). For the recruitment of patients, PCPs received a list with inclusion and exclusion criteria along with a screening list and a list of random numbers. PCPs were asked to create a list of all potentially eligible patients registered in their practice software which were enrolled in the DMP Diabetes; these patients were then registered in their practice software in 2013. From this list, PCPs selected patients according to the sequence indicated by the random numbers (provided by the study office). The randomly selected patients were checked for inclusion and exclusion criteria. Eligible patients were contacted and asked about their interest in participating in the study by their PCP physician. The procedure was repeated until a total of 20 patients per PCP were recruited. The number of patients screened and asked to participate was documented by the PCP physician and reported to the study office.

### Data collection

Patients were invited by the recruiting physician to fill out a pseudonymized paper-based questionnaire (self-administered by patients). This questionnaire captured the patient reported outcomes (e.g., German Version of the Summary of Diabetes Self-Care Activities Measure [29], socio-demographic aspects, and questions about utilization of health care within the last 9 months. Additionally, PCPs documented data from patients’ charts in a pseudonymized paper-based questionnaire (clinician administered; including inclusion/exclusion criteria, diagnoses, medication(s), hospitalizations, and hypoglycemia before recruitment) and assessed the clinical status of the patient (e.g., blood pressure, blood glucose, latest HbA1c value). The patient questionnaire and physician-reported chart-review were performed at baseline (T0) and after 9 months (01/11/2014 to 31/07/2015) (T1). Longer time frames for baseline (T0) and follow up (T1) were not feasible because of the limited length of the study. Participants were not provided with feedback following data collection points.

### Randomization

**Fig 1:**
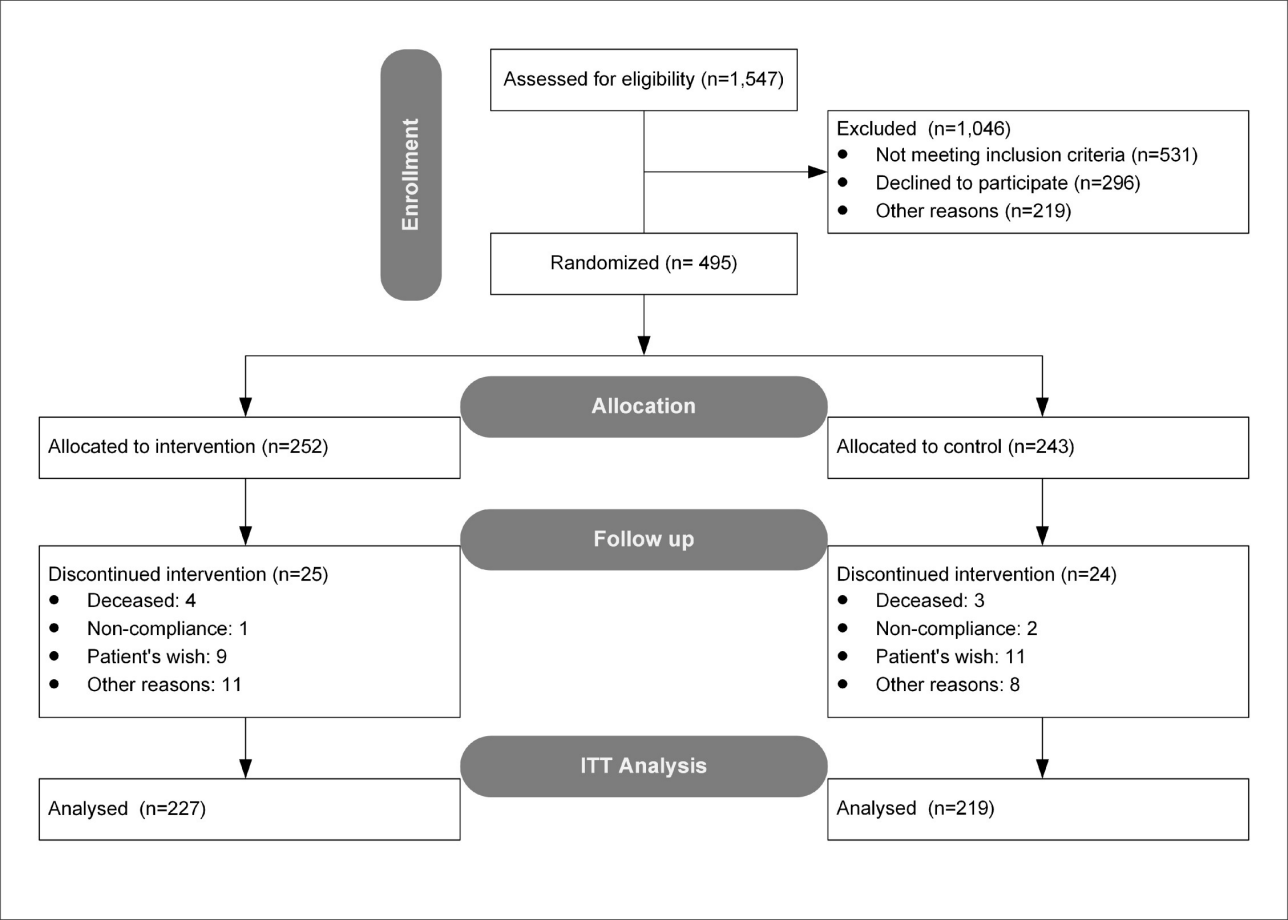
CONSORT flowchart.

After obtaining written informed consent, patients were randomized individually at the study office. Patients were randomly allocated by means of a randomization list, created by the trial statistician, to care management (intervention group) or usual care (control group) at the individual level at a ratio of 1:1. Then, patients were stratified by (1) type of medical treatment (insulin vs. oral medication or no medication) of their index disease (type 2 diabetes) and (2) study center (PCP) using block randomization with varying block lengths (4-4-2 scheme). The randomization was performed at the study office by a clinical monitor, who acted as sole randomization authority, within the used study-specific software. This person was not involved in the care of the trial patients or analysis of data. The clinical monitor allocated participants who had completed the self-administered and clinician administered questionnaire on a weekly basis to intervention or control groups using the randomization list that was provided by the Institute of Medical Biometry and Informatics (University Hospital Heidelberg). Therefore, the clinical monitor checked within the study-specific software if the written informed consent was available, inclusion criteria were fulfilled, and type of medical treatment before randomization. Allocation to intervention or control group were recorded in the software and patients allocated to intervention group were visible to the net care manager (NCM) within the software. Randomization was done at the patient level, so physicians provided care for patients in the intervention as well as control group. Blinding of physicians, medical assistants (MAs), and patients was not possible due to the nature of the intervention. All study results are reported according to CONSORT guidelines [42]. A flow diagram, as recommended in CONSORT is provided as Fig 1.

### Intervention design

#### Intervention conditions

This intervention was a care management program aimed at improving diabetes self-care behavior among patients with type 2 diabetes and multiple comorbidities. The development of the multifaceted intervention was based on a 12-month pilot study (GEDIMA), focus groups with PCP physicians and specialist care providers, and the active engagement of representatives of local patient self-help groups in formulating assessment contents and identifying community resources [36].

**Fig 2:**
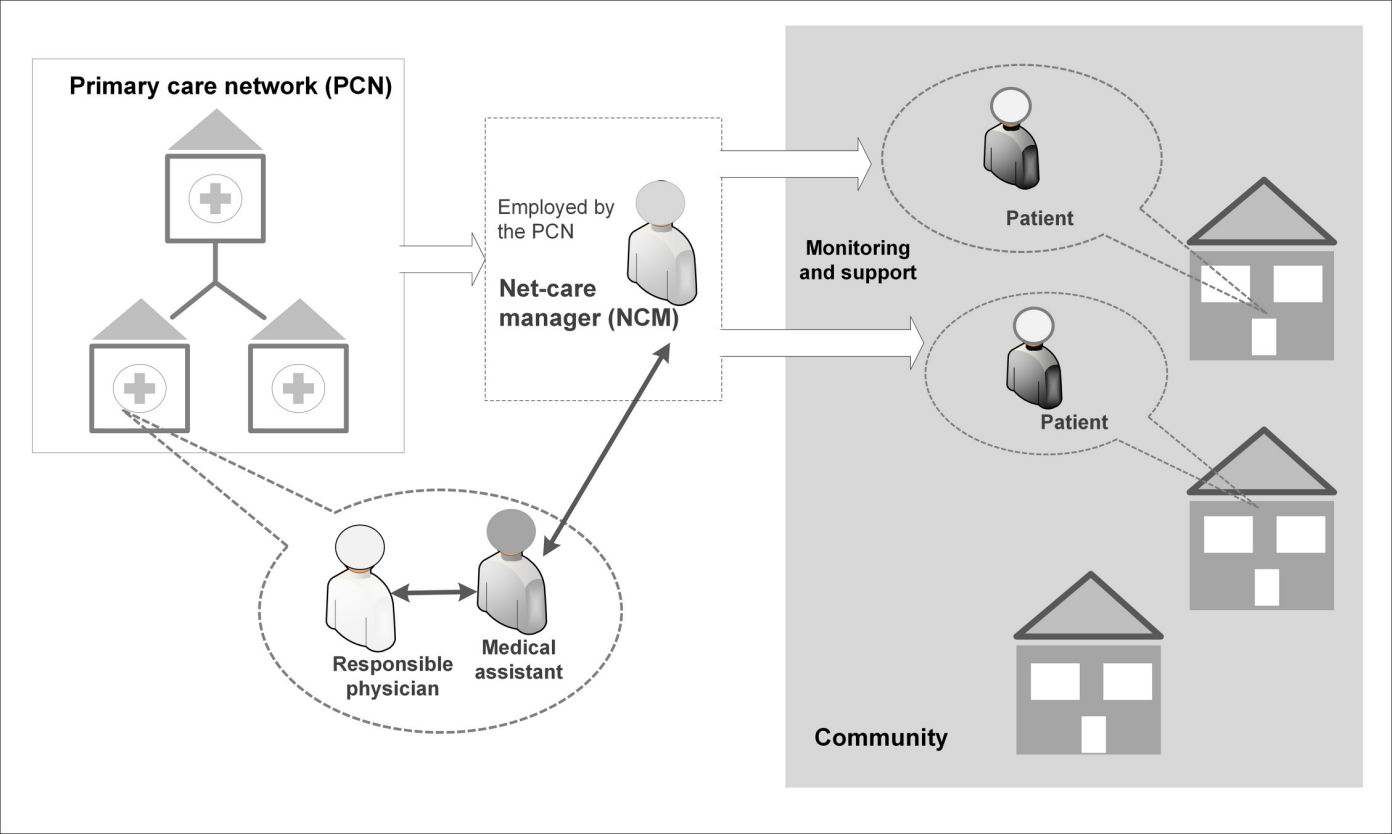
PCN-based care management.

Different to other care management interventions often implemented on practice or insurance levels, this intervention was embedded in a PCN. This means that the care manager, in this conceptual design called net-care manager (NCM), is employed by the PCP network and responsible for the care management of patients from different PCPs within the network. To ensure the continuity of care and the collaboration between involved health professionals, a medical assistant (MA) in each PCP worked as a link between the NCM and the responsible physician. This concept of shared resources is especially beneficial for smaller practices without their own resources for implementing care management approaches (Fig 2). Also, it facilitates shared learning and coordinated standardization of healthcare delivery across practices.

**Fig 3:**
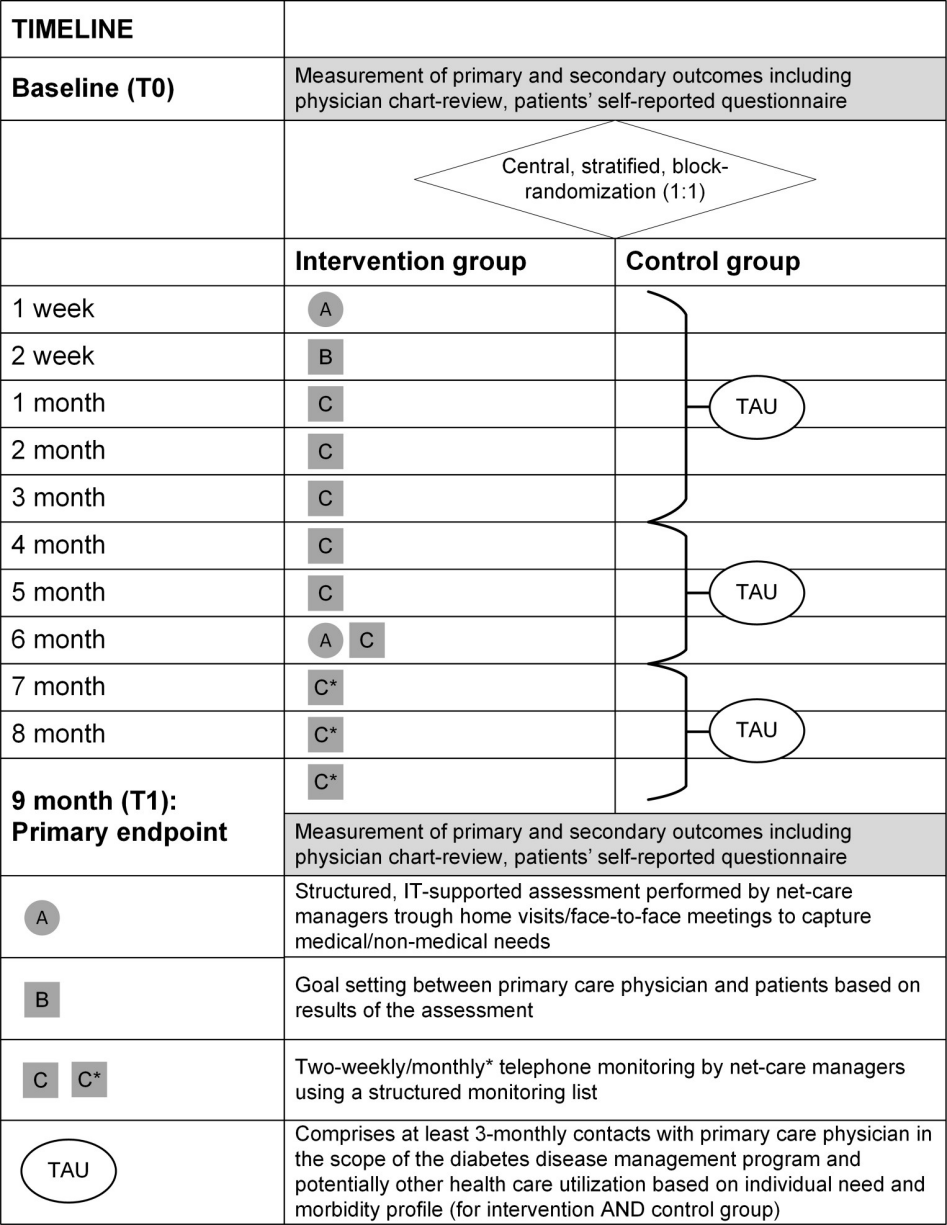
Timeline and care provided in both groups.

The care management intervention is an add-on to treatment as usual within the DMP for patients with type 2 diabetes and consists for those patients in the intervention group of an individualized IT-based assessment (e.g., health care and social aspects, for details see Bozorgmehr et al. 2014) [36], including home visits and telephone-monitoring on a regular basis, both provided by the NCM. Regarding the assessment, the NCM visits patients in the intervention group three times at home within the first weeks after entry to the study and after 6 months (Fig 3). The results of the assessment were reported to the responsible physician via the MA in each practice.

The objective of the telephone-monitoring was to detect acute clinical needs and to provide support related to self-care goals and healthcare related issues (e.g., medical or social problems). The monitoring was based on a structured list of questions, which was developed on the basis of experiences in prior projects of this PCP network. Additionally, to this structured monitoring, NCMs could support patients via telephone to locate additional services within the health system or the broader community according to patients’ individual preferences and needs. In total, 24 telephone-monitoring sessions were planned (every 2 weeks for a period of 6 months, once a month throughout the rest of the intervention period for patients in the intervention group) (36). No additional expenses were incurred for patients in the intervention group. Medical assistants providing the above described elements of the intervention to study participants had to participate in a group-based training comprising 32 hours before the intervention start.

#### Control conditions

Patients allocated to the control group received treatment as usual within the scope of the German Disease Management Program for Type 2 Diabetes mellitus. The German DMP for Type 2 Diabetes mellitus comprises (among other elements) evidence-based clinical guidelines, quarterly visits in primary care as well as eye and foot exams on a regular basis [5, 6].

### Primary and secondary outcomes

In this study, patient-level outcomes are of interest. In particular, the described intervention was aimed at improving self-care as a multidimensional construct consisting of the following five dimensions: diet, exercise, self-monitoring of blood glucose (SMBG) and foot care. This primary outcome was measured with the German version of the Summary of Diabetes Self-Care Activities Measure (SDSCA-G). This 11-item questionnaire has been shown to be a reliable and valid tool for assessing self-management in adults with type 2 diabetes in Germany [29]. The English version of the SDSCA is a widely used, valid and reliable tool for capturing self-care behavior [2, 37]. The SDSCA-G sum score was calculated as the mean of the first 10 items (not including the eleventh item measuring smoking behavior). The primary outcome for this study was the change in self-care behavior at month 9 compared to baseline.

As secondary outcomes, the mean change in each of the five dimensions of the SDSCA-G, the difference over time for the single SDSCA-G items and the HbA1c, the prevalence of (severe) hypoglycemia at T0 and T1 as well as the equity/efficacy ratio among the lowest vs. highest socioeconomic patient subgroups for the SDSCA-G score and HbA1c were considered.

### Sample size calculation

The sample size was calculated based on the expected difference between the two treatment groups in mean change in the SDSCA-G score from baseline (T0) compared to 9 months after baseline (T1). Based on data from published studies, which used the revised SDSCA as outcome measure [38–40], we estimated a mean change of 0.5 days (standard deviation 2.0) in the overall SDSCA-G score (calculated as the sum of days of items 1–10 divided by 10) per patient in 9 months as minimal clinically relevant change.

Based on these estimates, a total of 506 patients (253 per arm) would be required to detect an effect size (Cohen’s d) of 0.25 between-groups (intervention vs. control (50% relative increase in self-care) with a power of 80% applying a two-sided t-test for two independent samples at a significance level of 5%. It was assumed that analyzing the data using a linear mixed model including the fixed factor type of medical treatment, the fixed covariate baseline SDSCA-G, and the random factor NCM would lead to less unexplained variance and thus to an additionally increased power as compared to an analysis via two-sided t-test. Assuming a drop-out rate of 15% over the period of 9 months, the overall required sample size amounts to a total of 582 participants (291 per arm). No treatment by strata interaction was assumed in the sample size calculation, which was performed using ADDPLAN v6.0. (ICON, Leopardstown, Ireland). We did not conduct interim analyses and had no stopping guidelines.

### Statistical methods

The primary outcome was the change in SDSCA-G score from baseline (T0) to 9 months after baseline (T1), i.e., the difference SDSCA-G T1 –T0. The study objective was statistically formulated as a test of the null hypothesis H0: μ1 = μ2 (the mean difference SDSCA-G T1–T0 is equal in the two groups) against the alternative hypothesis H1: μ1 ≠ μ2 (the mean difference SDSCA-G T1–T0 is different in the two groups). The null hypothesis was tested at the two-sided significance level of α = 0.05. The primary analysis was carried out according to the intention-to-treat principle, i.e., all randomized subjects were included.

The difference SDSCA-G T1–T0 was described by treatment arm and in total. The number of cases, mean, standard deviation, median, interquartile range (IQR), minimum, and maximum were determined. Due to the hierarchical structure of the data, a multilevel analysis was performed with patients at level one and NCMs at level two. The primary (linear mixed) model with the SDSCA-G score difference T1 –T0 as the response variable include treatment group, type of medical treatment, and baseline SDSCA-G (T0) as fixed factors and NCM as random factor (i.e., NCM as random intercept) to account for the two-level data structure (patients nested in NCMs). The random intercept, as well as the residuals, was assumed to be normally distributed with variance $\sigma_{b}^{2}$ and $\sigma_{e}^{2}$, respectively. The results are presented as the mean between-group difference in SDSCA-G T1–T0 with the corresponding two-sided 95% confidence interval. The associated Cohen’s effect size d was calculated. To fit the primary model, as well as all models described in the next sections, the restricted maximum likelihood (REML) approach was used. Only the results from the primary analysis are to be interpreted in a confirmatory manner. Missing data in the primary outcome was addressed through the use of multiple imputation, taking the covariates intervention group and type of medical treatment into account by application of the fully conditional specification method [41]. This was realized using the option “FCS” of the SAS “MI” procedure, which is implemented in SAS v9.4 (SAS Institute, Carey, NC).

To support the primary analysis some sensitivity analyses were conducted, e.g., a complete case analysis, an analysis including an interaction term between type of medical treatment and intervention group, and analysis taking a number of comorbidities, age, and gender into account. Additionally, the SDSCA sum score and all subscores were assessed separately for the intervention and control group by using the complete case multi-level model’s least square means estimates, thus providing linear contrasts for treatment effects over time within each group. These models were also used to calculate intra-class correlation coefficients (ICC), measuring the amount of variance additionally explained by the two-level structure. As further secondary outcomes, the SDSCA items and the HbA1c difference over time (for the total patient population and for the subgroup with a baseline HbA1c below 7.5%) were analyzed analogously with two-level linear mixed models taking the respective baseline value and type of medical treatment into account. Furthermore, the prevalence of (severe) hypoglycemia at T0 and T1 was assessed descriptively by means of absolute and relative frequencies per group since. Also, the equity/efficacy ratio will be calculated as the ratio between the change (compared to T0 at T1) in SDSCA-G ‘score’ and HbA1c (%) between the lowest vs. highest socioeconomic patient subgroups (including education, income, degree, and migrant status), where confidence intervals for the ratios were determined using a bootstrap approach with 1,000,000 simulated datasets. Statistical analyses were carried out using SAS v9.4 (SAS Institute, Carey, NC) and IBM SPSS Statistics for Windows, version 25 (IBM Corp., Armonk, NY., USA).

## Results

No harms or unintended effects of this study have been reported within in the intervention group or the control group.

### Study enrollment and follow up

The recruitment of study patients took place in primary care practices within the GGM network between February and October 2014. Overall, a total of 1,541 patients were assessed for eligibility. Patients were excluded (n = 531) that did not meet the inclusion criteria; 296 patients declined to participate, and 219 patients were excluded for other reasons. The remaining 495 patients were randomized to intervention (*n* = 252) or control (*n* = 243). Two hundred and twenty-seven (90.1%) patients in the intervention and 219 (90.1%) patients in the control group remained in the trial until follow-up, leaving an arrition rate of 9.9% in both study groups. The per protocol (PP) set comprised 119 patients in the intervention and 219 patients in the control group.

### Characteristics of patients

In the intervention group, 47.2% were female, the mean age ± SD was 68 ± 11 years, and the mean Body Mass Index (BMI) was 31.5±6.7 kg/m^2^. The diabetes duration was 14.1 ± 9.5 years, and the HbA1c was 7.1 ± 1.2% (148.1 ± 47.6 mg/dl) on average. Of all patients in the intervention group, 33.7% received treatment with insulin. On average, every patient had 3.8 additional conditions (Table 1).

**Table 1:** Patient characteristics—comparison between groups at baseline.

	Intervention	N	Control	N
**Number**		252		243
**Age years (SD)**	68.40 (11.33)	249	68.31 (10.80)	238
**Female**	47.22%	119	48.56%	118
**Married / cohabited**	58.73%	148	53.09%	129
**Up to 9 years in school**	30.16%	76	26.75%	65
Professional education*	66.67%	168	67.07%	163
**Diabetes duration in years (SD)**	14.13 (9.50)	187	15.06 (12.07)	173
**Treatment with Insulin**	33.7%	85	33.3%	81
**Enrolled in an additional DMP**^**$**^	23.8%	60	28.0%	68
**Number of Comorbidities**	3.79 (1.16)	252	3.74 (1.22)	243
**Mobility restrictions**	18.7%	47	17.3%	42
**Hospital stays per patients**^**#**^	0.19 (0.51)	252	0.23 (0.61)	243
**Systolic blood pressure (mmHg)**	135.45 (14.65)	252	133.88 (14.25)	243
**Diastolic blood pressure (mmHg)**	80.27 (8.84)	252	79.63 (8.59)	243
**Blood glucose (mg/dl)**	148.11 (47.56)	252	151.76 (50.13)	243
**Blood glucose (HbA1C)**	7.13 (1.23)	252	7.25 (1.19)	243
**BMI calculated**	31.52 (6.73)	252	31.62 (5.97)	243

*minimum vocational training

^$^Asthma, CHD, COPD

^#^last 9 month

In the control group, 48.5% were female, the mean age ± SD was 68 ± 11 years, and the mean BMI was 31.6±6.0 kg/m^2^. The diabetes duration was 15.1 ± 12.1 years, and the HbA1c was 7.3 ± 1.2% (151.8 ± 50.1 mg/dl) on average. Of all patients in the intervention group, 33.3% received treatment with insulin. On average, every patient had 3.7 additional conditions (Table 1).

### Characteristics of physicians, medical assistants, and net case manager

The mean age ± SD of participating physicians (n = 32; 45.2% female) was 54.6 ± 9.3 years old. Their average work experience ± SD was 25.9 ± 8.8 years. Of all participating practices, 66.7% were single-handed. Regarding MAs (N = 23; 100.0% female) and NCMs (N = 11; 100.0% female) their mean age ± SD was 43.5 ± 12.1 years and 42.7 ± 9.5 years respectively. The mean work experience ± SD of MAs was 21.4 ± 12.4 years and for NCMs 22.3 ± 9.8 years (Table 2).

**Table 2:** Characteristics of physicians, medical assistants, and net case manager (NCM).

	Physicians		N	MA		N	NCM		N
**Age years (mean, SD)**	54.6	(9.3)	32	43.5	(12.1)	23	42.7	(9.51)	11
**Female (percent)**	45.2%		14	100.0%		23	100.0%		11
**Years of work experience (mean, SD)**	25.9	(8.8)	30	21.4	(12.4)	23	22.3	(9.8)	11

### Descriptive analysis

At baseline (T0) valid sum scores for 368 patients were available (intervention (I) = 186; control (C) = 182) and the mean SDSCA sum score in the intervention group was 3.31 +/-1.11 vs. 3.50 +/-1.23 in the control group. After 9 months, valid sum scores for 345 patients were available (I = 181; C = 164) and the mean SDSCA sum score sample in the intervention group was 3.63 +/-1.22 vs. 3.58 +/-1.22 in the control group. More detailed information is available in the appendix (S1 Appendix, Table B).

The analysis of differences over time within treatment groups has shown significantly increased values from T0 to T1 within the intervention group for the SDSCA sum score (p = 0.012). Within the control group, none of the scores have changed significantly (Table 3).

**Table 3:** Description of SDSCA scores within the intervention and control group alongside p-values for differences in scores over time.

SDSCA-G	Intervention							Control group						
	T0			T1			P**							P**
	N	Mean	SD	N	Mean	SD		N	Mean	SD	N	Mean	SD	
**sum**	186	3.31	1.11	181	3.63	1.22	0.012	182	3.50	1.23	164	3.58	1.22	0.197
**diet**	208	4.30	1.58	199	4.54	1.50	0.180	201	4.38	1.76	186	4.40	1.72	0.959
**exercise**	231	3.13	1.84	209	3.12	1.83	0.876	220	3.01	1.85	194	3.10	2.01	0.731
BST*	78	5.48	2.28	73	5.58	2.01	0.781	75	5.45	2.26	68	5.93	1.90	0.051
**foot care**	222	2.47	2.19	211	2.85	2.28	0.207	223	3.06	2.25	195	3.15	2.46	0.322

*blood sugar test

**p-value for testing whether least square means are equal to 0, based on linear mixed model with repeated measurements

### Primary outcome

The primary analysis showed an intervention effect of 0.14 (intervention–control) for the difference in SDSCA sum score between T1 and T0, which was, however, not statistically significant (p = 0.2063, 95%-CI = [-0.0838; 0.3844], Cohen’s d = 0.1597). Sensitivity analyses yielded results that were in line with this finding (Table 4). The ICC amounted to 2.3%, thus, a small, but nonetheless existent dependency between patients assigned to the same NCM was observed.

**Table 4:** Primary outcome.

Model	Estimate	95%-CI	Cohen’s d	p-value
**Primary model** (adjusted for multilevel structure and covariates, multiple imputation on score level, ITT)	0.1503	[-0.0838; 0.3844]	0.1597	0.2063
**Sensitivity analysis** of primary model (adjusted for multilevel structure and covariates, no imputation, ITT)	0.1151	[-0.0927; 0.3229]	0.1223	0.2764
**Sensitivity analysis** of primary model (adjusted for multilevel structure covariates, and interaction between type of medical treatment and intervention group, no imputation, ITT)	0.1246	[-0.0918; 0.3409]	0.1326	0.2580
**Sensitivity analysis** of primary model (adjusted for multilevel structure and covariates, no imputation, PP)	0.1517	[-0.1056; 0.4087]	0.1617	0.2466
**Sensitivity analysis** of primary model by two-sided t-test (unadjusted for multilevel structure, unadjusted for covariates, no imputation, PP)	0.1858	[-0.0381; 0.4098]	0.2311	0.1035

### Secondary outcomes

The analysis of secondary outcomes has shown no significant change for the sub-score diet (p = 0.1385, 95%-CI = [-0.0591; 0.4229], Cohen’s d = 0.1444), exercise (p = 0.7137, 95%-CI = [-0.2658; 0.3878], Cohen’s d = 0.0343), blood glucose testing (p = 0.2227, 95%-CI = [-0.8750; 0.2057], Cohen’s d = -0.1789), and foot care (p = 0.8472, 95%-CI = [-0.3482; 0.4239], Cohen’s d = 0.0185) (Table 5). However, the analysis of the difference over time for the single SDSCA-G items (S1 Appendix, Table C) and the HbA1c (S1 Appendix, Table D), the prevalence of (severe) hypoglycemia at T0 and T1 (S1 Appendix, Table E) as well as the analysis of equity/efficacy ratio (S1 Appendix, Table F) haven’t shown noteworthy results.

**Table 5:** SDSCA-G sub-scores*.

	Estimate	95%-CI	Cohen’s d	p-value
Diet	0.1819	[-0.0591; 0.4229]	0.1444	0.1385
Exercise	0.0610	[-0.2658; 0.3878]	0.0343	0.7137
Blood glucose testing**	-0.3346	[-0.8750; 0.2057]	-0.1789	0.2227
Foot care	0.0379	[-0.3482; 0.4239]	0.0185	0.8472

*ITT analysis (adjusted for multilevel structure and covariates)

**Insulin patients only

### Intervention fidelity

Overall, the fidelity of the intervention was low. Only 119 patients got the intervention as intended (per protocol; 2 home visits and 10 telephone-monitoring calls). Regarding telephone-monitoring, only 6 (2.6%) patients received all planned 15 calls, whereas 1 patient (0.4%) never received even one call. The majority of patients (83.3%) received between 8 and 14 calls within the intervention period (T0 to T1). Additionally, 190 patients (83.7%) got 2 home visits until T1 as proposed.

Differences between the intended and the implemented intervention also exist for recruiting and the enrollment of patients to NCMs. Concerning recruiting, 16 physicians (47.1%) reached the recruitment goal of 20 patients. More than 10 patients were recruited by 8 physicians (23.5%). In additon, the number of patients enrolled to each NCM in the intervention group varied between 13 patients per NCM and 31 patients per NCM.

## Discussion

This study aimed to assess the effectiveness of a PCN based, IT-supported care management intervention with integrated telephone monitoring for the improvement of self-care behavior among patients with type 2 diabetes mellitus and multimorbidity. The results of our primary analysis showed no statistically significant effect. The sum score for self-care behavior increased significantly in the intervention group over time, but not in the control group. Additionally, significant changes between T0 and T1 were observed in the intervention group for the subscores on healthy diet and foot care.

Previous studies have often either focused on strengthening self-management or the implementation of care management interventions. Whereas self-management education and training are effective [43–45], most care management programs in the US for type 2 diabetes are carved-out, accomplish limited effects on metabolic outcomes, and have indefinite effects on relevant patient outcomes such as health-related quality of life [46]. More recently, the implementation of care management tools like home visits and telephone-based intervention have shown promising results in improving self-care in patients with type 2 diabetes [23, 47–49].

However, none of those interventions addressed multimorbid patients and implementation in primary care remains scarce [36]. The lack of resources in primary care practices to implement care management programs might be a reason for this lack. Especially in chronic care, comprehensive and interprofessional approaches are urgently needed to address diabetes and comorbidities. PCNs could offer the necessary infrastructure and previous research has shown that diabetes care in PCNs is associated with more frequent guideline-recommended screening and a lower rate of admissions to hospitals or visits to emergency departments [30].

Nevertheless, strengthening self-care in patients with comorbidities is still a challenge. As shown by the study of Bos-Touwen et al. (2015), the burden of disease (e.g., regarding comorbidities) and disease duration are associated with poor activation for self-management [50]. Moreover, compliance or adherence to self-care activities are often low, particularly regarding long-term changes. Multiple demographic, socio-economic and social support related aspects play a critical role in self-management and have to be considered in facilitating self-care activities in patients with diabetes [3].

Previous research indicates that, in particular, patients with suboptimal or poor glycemic control receive a greater benefit from self-management programs [51]. On the other hand, controlling blood sugar is well implemented in the German DMP for Type 2 Diabetes [52]. Accordingly, in our study population, the HbA1C was modest for both groups. This could be one reason why no larger effects were observed in our study. Another reason could be that the quality of care in diabetes patients is already high. Besides the impact of the German DMP [52–54], for example, the study of Nouwens et al. (2012) has shown that the presence of diabetes is associated with better preventative treatment of cardiovascular risk factors [55].

Additionally, the implementation of complex interventions in healthcare practice is often challenging [56], resulting in suboptimal delivery of the planned interventions. Low intervention fidelity is one of the potential explanations for the lack of effect in this trial. This is particularly relevant for intervention components, which have been shown to be effective in previous research. Examples are medical assistant-based telephone monitoring or the implementation of the intervention in primary care practices [26, 28].

Regarding future development in this area, there are important lessons we have learned with this study. First, PCN-based care management interventions have the potential to improve self-care behavior among multimorbid patients with type 2 diabetes. To reach this aim, future implementations should focus on intervention fidelity and the improvement of intervention elements.

In particular, a stronger definition of the concept for home visits and the telephone-monitoring seems to be appropriate. Although there were guidelines in our study, the individual implementation was strongly dependent on the specific NCM. The integration of motivational interviewing strategies could be helpful to improve both the focus and the results of the NCM intervention. As shown by Masterson et al. (2016) in patients with chronic heart failure, motivational interviewing used during home visits and follow up calls can lead to significant and clinically meaningful improvements in self-care maintenance [57].

Too, it is important to improve the process of communication between the NCM, the medical assistant, and the treating physician (see Fig 1). In our study, the treating physicians were not always well informed about the results of the home visits and telephone calls done by the NCM. This is problematic because the role of physicians in promoting self-care is vital. In particular, trust seems to be important. The study of Bonds et al. (2004) indicates that higher patient trust in physicians is associated with reduced patient difficulty in completing disease-specific tasks [58].

Finally, this study was focused on the individual patient. Further development of this PCN-based care management approach could involve the social network of patients to a greater extent. Since primary care physicians often provide care to multiple family members, they are ideally positioned to expand this intervention [56] to include patient support networks. Additionally, community and social support networks operating in patients’ lives should be more engaged [59].

### Strength and limitations

To the best of our knowledge, this study is the largest RCT in primary care to evaluate a care-management intervention aimed at improving self-care. The implementation in a PCN and the focus of multimorbid patients with type 2 diabetes are also highly innovative. The collection of relevant data for the analysis was done with well-established measures to generate highly valid data. In addition, biomedical outcomes such as laboratory measures and chronic conditions used in this study were determined by healthcare professionals, rather than being self-reported by patients. The multi-level approach used in this study is seen as relevant in health services research due to the hierarchical structure of the patients’ data clustered in PCP-teams [60]. However, the primary analysis showed no statistically significant effect and the recruitment target was not achieved. Instead of 600 patients, we were able to recruit 495 patients. Recruitment of patients was not stopped actively. However, participating study centers were not able to enroll additional eligible patients. Of these 495 patients, only 448 completed follow-up, which was below the calculated net sample size of 506 required to detect the assumed treatment effect of d = 0.25 with a t-test; this resulted in a slight underpowering of the study (achieved power with n = 448 patients amounts to only 75.18% under the same assumptions). Moreover, due to the randomization on patient level there was an increased risk of contamination of intervention effects. Regarding the implementation, the intervention fidelity in this study was moderate and we used a convenience sample of PCP-teams in only one PCN. The motivation of the participating PCP-teams may not be comparable to PCP-teams in general. Therefore, the generalizability of the findings is limited.

### Conclusions

The results of our primary analysis showed no statistically significant effect. Otherwise, the sum score for self-care behavior increased significantly in the intervention group over time, but not in the control group. Possible reasons are the high baseline performance in our sample and the low intervention fidelity. The future development of this approach should focus on a closer definition of the intervention, improvements regarding the communication between the NCM and the treating practice, as well as an increased engagement of the social network. Especially for smaller primary care practices, the implementation of care management in PCNs may improve the self-care behavior of patients.

## Supporting information

S1 Appendix (DOCX)

S1 CONSORT 2010 Checklist (PDF)

S1 Study Protocol (PDF)

## Abbreviations

DMP: Disease management program
HRQoL: Health-related quality of life
PCP: Primary care practices
PCN: Primary care network
RCT: Randomized controlled trial
GGM: Genossenschaft Gesundheitsprojekt Mannheim
NCM: Net-care manager
GEDIMA: [Gesundheitsbegleitung Diabetes Mannheim]
SMBG: Self-monitoring of blood glucose
SDSCA-G: Summary of diabetes self-care activities measure, German version
IQR: Interquartile range
ICC: Intraclass correlation
REML: Restricted maximum likelihood
PP: Per protocol
ITT: Intention to treat
