# Correction: Care management intervention to strengthen self-care of multimorbid patients with type 2 diabetes in a German primary care network: A randomized controlled trial

**DOI:** 10.1371/journal.pone.0221229

**Published:** 2019-08-09

**Authors:** Dominik Ose, Martina Kamradt, Marion Kiel, Tobias Freund, Werner Besier, Manfred Mayer, Johannes Krisam, Michel Wensing, Hans-Joachim Salize, Joachim Szecsenyi

There is an error in Table 1. The letter “c” in the “Number of comorbidities” row should not be capitalized. Please see the correct Table 1 here.

**Table 1:** Patient characteristics—Comparison between groups at baseline.

	Intervention	N	Control	N
**Number**		252		243
**Age years (SD)**	68.40 (11.33)	249	68.31 (10.80)	238
**Female**	47.22%	119	48.56%	118
**Married / cohabited**	58.73%	148	53.09%	129
**Up to 9 years in school**	30.16%	76	26.75%	65
Professional education*	66.67%	168	67.07%	163
**Diabetes duration in years (SD)**	14.13 (9.50)	187	15.06 (12.07)	173
**Treatment with insulin**	33.7%	85	33.3%	81
**Enrolled in an additional DMP**^$^	23.8%	60	28.0%	68
**Number of comorbidities**	3.79 (1.16)	252	3.74 (1.22)	243
**Mobility restrictions**	18.7%	47	17.3%	42
**Hospital stays per patients**^#^	0.19 (0.51)	252	0.23 (0.61)	243
**Systolic blood pressure (mmHg)**	135.45 (14.65)	252	133.88 (14.25)	243
**Diastolic blood pressure (mmHg)**	80.27 (8.84)	252	79.63 (8.59)	243
**Blood glucose (mg/dl)**	148.11 (47.56)	252	151.76 (50.13)	243
**Blood glucose (HbA1C)**	7.13 (1.23)	252	7.25 (1.19)	243
**BMI calculated**	31.52 (6.73)	252	31.62 (5.97)	243

*minimum vocational training;

^$^Asthma, CHD, COPD;

^#^last 9 month;

There is an error in Table 3. The subheadings “T0” and “T1” are missing from the “Control group” section. Please see the correct Table 3 here.

**Table 3:** Description of SDSCA scores within the intervention and control group alongside p-values for differences in scores over time.

SDSCA-G	Intervention							Control group						
	T0			T1			P**	T0			T1			P**
	N	Mean	SD	N	Mean	SD		N	Mean	SD	N	Mean	SD	
**sum**	186	3.31	1.11	181	3.63	1.22	0.012	182	3.50	1.23	164	3.58	1.22	0.197
**diet**	208	4.30	1.58	199	4.54	1.50	0.180	201	4.38	1.76	186	4.40	1.72	0.959
**exercise**	231	3.13	1.84	209	3.12	1.83	0.876	220	3.01	1.85	194	3.10	2.01	0.731
BST*	78	5.48	2.28	73	5.58	2.01	0.781	75	5.45	2.26	68	5.93	1.90	0.051
**foot care**	222	2.47	2.19	211	2.85	2.28	0.207	223	3.06	2.25	195	3.15	2.46	0.322

*blood sugar test;

**p-value for testing whether least square means are equal to 0, based on linear mixed model with repeated measurements

There is an error in reference 32. The correct reference is: Sheaff R, Gené-Badia J, Marshall M, Sˇvab I. The evolving public-private mix. In: Saltman RB, Rico A, Boerma W, Hrsg. Primary care in the driver’s seat? Organizational reform in European primary care. New York City, NY: Open University Press; 2006. S. 129–46.

Within references 1, 5, 15, 20, 26, 29, 31, 34, 40, 47, 49, and 54, the “View Article” links do not lead to the cited articles and some PMIDs are not listed. Please view the complete references here.

1. Korff M von, Gruman J, Schaefer J, Curry SJ, Wagner EH. Collaborative management of chronic illness. Ann Intern Med 1997; 127(12):1097–102. https://doi.org/10.7326/0003-4819-127-12-199712150-00008 PMID:9412313

5. Busse R. Disease management programs in Germany’s statutory health insurance system. Health Aff (Millwood) 2004; 23(3):56–67. https://doi.org/10.1377/hlthaff.23.3.56 PMID:15160803

15. Vogeli C, Shields AE, Lee TA, Gibson TB, Marder WD, Weiss KB et al. Multiple chronic conditions: prevalence, health consequences, and implications for quality, care management, and costs. J Gen Intern Med 2007; 22 Suppl 3:391–5. https://doi.org/10.1007/s11606-007-0322-1 PMID:18026807

20. Wagner EH, Austin BT, Davis C, Hindmarsh M, Schaefer J, Bonomi A. Improving chronic illness care: translating evidence into action. Health Aff (Millwood) 2001; 20(6):64–78. https://doi.org/10.1377/hlthaff.20.6.64 PMID:11816692

26. Gensichen J, Korff M von, Peitz M, Muth C, Beyer M, Guthlin C et al. Case management for depression by health care assistants in small primary care practices: a cluster randomized trial. Ann Intern Med 2009; 151(6):369–78. https://doi.org/10.7326/0003-4819-151-6-200909150-00001 PMID:19755362

29. Kamradt M, Bozorgmehr K, Krisam J, Freund T, Kiel M, Qreini M et al. Assessing self-management in patients with diabetes mellitus type 2 in Germany: validation of a German version of the Summary of Diabetes Self-Care Activities measure (SDSCA-G). Health Qual Life Outcomes 2014; 12(1):185. https://doi.org/10.1186/s12955-014-0185-1 PMID:25519204

31. Amelung V, Hildebrandt H, Wolf S. Integrated care in Germany-a stony but necessary road! Int J Integr Care 2012; 12:e16. https://doi.org/10.5334/ijic.853 PMID:22977429

34. Eisenstat SA, Chang Y, Porneala BC, Geagan E, Wilkins G, Chase Bet al. Development and Implementation of a Collaborative Team Care Model for Effective Insulin Use in an Academic Medical Center Primary Care Network. Am J Med Qual 2016. https://doi.org/10.1177/1062860616651715 PMID:27259871

40. Lee H, Ahn S, Kim Y. Self-care, Self-efficacy, and Glycemic Control of Koreans With Diabetes Mellitus. Asian Nurs Res (Korean Soc Nurs Sci) 2009; 3(3):139–46. https://doi.org/10.1016/S1976-1317(09)60025-6 PMID:25030472

47. Collins AE, Niles BL, Mori DL, Silberbogen AK, Seligowski AV. A telephone-based intervention to promote diabetes management in veterans with posttraumatic stress symptoms. Prof Psychol Res Pr 2014; 45(1):20–6. https://doi.org/10.1037/a0032604

49. Sarayani A, Mashayekhi M, Nosrati M, Jahangard-Rafsanjani Z, Javadi M, Saadat N et al. Efficacy of a telephone-based intervention among patients with type-2 diabetes; a randomized controlled trial in pharmacy practice. Int J Clin Pharm 2018. https://doi.org/10.1007/s11096-018-0593-0 PMID:29435911

54. Ose D, Miksch A, Urban E, Natanzon I, Szecsenyi J, Kunz CU et al. Health related quality of life and comorbidity. A descriptive analysis comparing EQ-5D dimensions of patients in the German disease management program for type 2 diabetes and patients in routine care. BMC Health Serv Res 2011; 11(1):179. https://doi.org/10.1186/1472-6963-11-179 PMID:21810241

## References

[1] Ose D, Kamradt M, Kiel M, Freund T, Besier W, Mayer M, et al (2019) Care management intervention to strengthen self-care of multimorbid patients with type 2 diabetes in a German primary care network: A randomized controlled trial. PLoS ONE 14(6): e0214056 10.1371/journal.pone.0214056 31188825 PMC6561631

