# Supplementary material for: Care management intervention to strengthen self-care of multimorbid patients with type 2 diabetes in a German primary care network: A randomized controlled trial
Source: PLoS One. 2019 Jun 12;14(6):e0214056. doi: 10.1371/journal.pone.0214056 (PMC6561631; doi:10.1371/journal.pone.0214056)
Supplement: S1 Appendix — (DOCX) [file pone.0214056.s001.docx]

**Table A:** Description of SDSCA SUM score (ITT)*

| **Variable** | **Intervention (N=252)** | **Control (N=243)** |
| --- | --- | --- |
| **SDSCA Sum score (T0)** | | |
| N | 186 | 182 |
| Mean +/- SD | 3.31 +/-1.11 | 3.50 +/-1.23 |
| Median | 3.30 | 3.35 |
| p25, p75 | 2.50, 4.00 | 2.60, 4.40 |
| Min, Max | 0.90, 6.20 | 0.60, 6.70 |
| **SDSCA Sum score (T1)** | | |
| N | 181 | 164 |
| Mean +/- SD | 3.63 +/-1.22 | 3.58 +/-1.22 |
| Median | 3.60 | 3.50 |
| p25, p75 | 2.80, 4.60 | 2.80, 4.50 |
| Min, Max | 0.60, 6.40 | 0.50, 7.00 |
| **SDSCA Sum score (T1-T0)** | | |
| N | 139 | 135 |
| Mean +/- SD | 0.23 +/-0.96 | 0.04 +/-0.92 |
| Median | 0.30 | 0.00 |
| p25, p75 | -0.20, 0.70 | -0.50, 0.60 |
| Min, Max | -2.90, 3.30 | -2.30, 2.40 |

*Complete SDSCA-Questionnaires only

**Table B:** Description of SDSCA SUM score (PP)*

| **Variable** | **Intervention (N=119)** | **Control (N=219)** |
| --- | --- | --- |
| **SDSCA Sum score (T0)** |  |  |
| N | 89 | 164 |
| Mean +/- SD | 3.46 +/-1.13 | 3.49 +/-1.23 |
| Median | 3.50 | 3.35 |
| p25, p75 | 2.80, 4.20 | 2.60, 4.35 |
| Min, Max | 0.90, 6.20 | 0.60, 6.70 |
| **SDSCA Sum score (T1)** |  |  |
| N | 98 | 164 |
| Mean +/- SD | 3.64 +/-1.20 | 3.58 +/-1.22 |
| Median | 3.65 | 3.50 |
| p25, p75 | 2.90, 4.60 | 2.80, 4.50 |
| Min, Max | 0.80, 5.80 | 0.50, 7.00 |
| **SDSCA Sum score (T1-T0)** |  |  |
| N | 78 | 135 |
| Mean +/- SD | 0.26 +/-0.97 | 0.04 +/-0.92 |
| Median | 0.40 | 0.00 |
| p25, p75 | -0.20, 0.80 | -0.50, 0.60 |
| Min, Max | -2.90, 2.90 | -2.30, 2.40 |

***** Complete SDSCA-Questionnaires only

**Table C**: Difference over time (T0 to T1) for the single SDSCA-G items

|  | **Estimate** | **95%-CI** | **Cohen’s d** | **p-value** |
| --- | --- | --- | --- | --- |
| SDSCA Item 1 | 0.0956 | [-0.2423; 0.4334] | 0.0503 | 0.5784 |
| SDSCA Item 2 | 0.0979 | [-0.2245; 0.4202] | 0.0544 | 0.5508 |
| SDSCA Item 3 | 0.1505 | [-0.2698; 0.5708] | 0.0604 | 0.4818 |
| SDSCA Item 4 | 0.4084 | [0.0223; 0.7944] | 0.1753 | 0.0382 |
| SDSCA Item 5 | 0.0758 | [-0.3347; 0.4862] | 0.0313 | 0.7168 |
| SDSCA Item 6 | 0.0266 | [-0.3442; 0.3974] | 0.0129 | 0.8879 |
| SDSCA Item 7** | -0.1578 | [-0.6820; 0.3665] | -0.0839 | 0.5527 |
| SDSCA Item 8** | -0.5796 | [-1.2473; 0.0881] | -0.2446 | 0.0883 |
| SDSCA Item 9 | 0.1719 | [-0.2620; 0.6059] | 0.0685 | 0.4365 |
| SDSCA Item 10 | -0.2138 | [-0.6642; 0.2366] | -0.0855 | 0.3513 |

*****ITT analysis (adjusted for multilevel structure and covariates); **Insulin patients only;

**Table D:** Physician-reported HbA1c (%) at T1 compared to T0

| **Secondary outcome*** | **Estimate** | **95%-CI** | **Cohen’s d** | **p-value** |
| --- | --- | --- | --- | --- |
| HbA1c (%) | -0.0043 | [-0.1854; 0.1768] | -0.0042 | 0.9626 |
| HbA1c (low subgroup, %)** | 0.0257 | [-0.1793;0.2308] | 0.0279 | 0.8052 |

*****ITT analysis (adjusted for multilevel structure and covariates)
**subgroup analysis based only on those patients with a baseline HbA1c less than 7.5%

**Appendix E:** Presence of physician-reported (severe) hypoglycemia among those patients at risk for this event during the respective observation period

| **Variable** | **Intervention (N=252)** | **Control (N=243)** |
| --- | --- | --- |
| Presence of hypoglycemia (T0) |  |  |
| - no | 100 (95.2%) | 108 (94.7%) |
| - yes | 5 (4.8%) | 6 (5.3%) |
| - missing | 147 | 129 |
| Presence of hypoglycemia (T1) |  |  |
| - no | 106 (95.5%) | 99 (97.1%) |
| - yes | 5 (4.5%) | 3 (2.9%) |
| - missing | 141 | 141 |
| Presence of severe hypoglycemia (T0) |  |  |
| - no | 102 (100.0%) | 109 (99.1%) |
| - yes | 0 (0.0%) | 1 (0.9%) |
| - missing | 150 | 133 |
| Presence of severe hypoglycemia (T1) |  |  |
| - no | 107 (99.1%) | 99 (98.0%) |
| - yes | 1 (0.9%) | 2 (2.0%) |
| - missing | 144 | 142 |

**Appendix F:** Equity/efficacy ratios for selected socioeconomic subgroups

| **Outcome** | **Subgroup 1** | **Subgroup 2** | **Equity/efficacy ratio  (Subgroup 1/ Subgroup 2) with 95% CI*** | |
| --- | --- | --- | --- | --- |
|  |  |  | **Treatment** | **Control** |
| **SDSCA sum score** | Low education  (9 years or less) | High education  (10 years or more) | 0.203/0.140=1.454  [-13.146; 15.087] | 0.008/0.25=0.031  [-2.180; 2.332] |
|  | Monthly income less than 1250 € | Monthly income higher than 4000 € | -0.195/0.226=-0.864  [-7.881; 7.471] | 0.003/0.061=0.051  [-5.361; 5.33] |
|  | No job-qualifying degree | University degree | 0.177/0.207=0.856  [-9.148; 10.171] | -0.242/0.230=-1.052  [-10.937; 9.918] |
|  | Migration background | No Migration background | 0.157/0.176=0.892  [-3.362; 7.962] | -0.118/0.104=-1.131 [-17.311; 14.978] |
| **HbA1c (%)** | Low education  (9 years or less) | High education  (10 years or more) | -0.022/0.140=-0.156  [-5.557; 5.220] | 0.081/-0.063=-1.291  [-10.661; 10.377] |
|  | Monthly income less than 1250 € | Monthly income higher than 4000 € | 0.161/0.187=0.861  [-6.728; 6.989] | 0.324/-0.538=-0.602  [-4.176; 1.291] |
|  | No job-qualifying degree | University degree | -0.083/0.153=-0.545  [-8.744; 8.135] | 0.171/-0.283=-0.604  [-7.217; 5.931] |
|  | Migration background | No Migration background | -0.225/0.126=-1.795  [-17.438; 12.433] | -0.01/0.088=-0.111  [-15.162; 15.155] |
| **HbA1c (HbA1c low subgroup, %)***** | Low education  (9 years or less) | High education  (10 years or more) | 0.067/0.372=0.179  [-0.398; 1.029] | 0.200/0.033=6.141  [-20.610; 21.089] |
|  | Monthly income less than 1250 € | Monthly income higher than 4000 € | 0.287/0.491=0.586  [-2.479; 4.654] | 0.324/-0.025=-12.719  [-17.862; 17.829] |
|  | No job-qualifying degree | University degree | 0.070/0.261=0.268  [-5.336; 6.015] | 0.218/0.046=4.767  [-14.258; 14.367] |
|  | Migration background | No Migration background | 0.041/0.245=0.169  [-1.45; 2.095] | -0.077/0.229=-0.334  [-3.255; 1.869] |

*Confidence intervals for ratios were determined using 1,000,000 bootstrapped samples; *** subgroup analysis based only on those patients with a baseline HbA1c less than 7.5%
