# Supplementary material for: Care management intervention to strengthen self-care of multimorbid patients with type 2 diabetes in a German primary care network: A randomized controlled trial
Source: PLoS One. 2019 Jun 12;14(6):e0214056. doi: 10.1371/journal.pone.0214056 (PMC6561631; doi:10.1371/journal.pone.0214056)
Supplement: S1 Study Protocol — (PDF) [file pone.0214056.s003.pdf]

UniversitätsKlinikum Heidelberg

Study Protocol

For submission to the ethics committee of the Medical Faculty Heidelberg

Title:
A multi-center, individual-level randomized, 18-month, parallel-group superiority trial to compare the efficacy of a complex primary care practice network-based care management intervention versus usual care in the improvement of self-care behavior among patients with type II diabetes and multiple comorbidities (GEDIMAplus)

Funding Agency:
Federal Ministry for Education and Research (BMBF)
In the scope of the project
"Gesundheitsregion der Zukunft"
- Subproject P7 -

Principal Investigator:

Prof. Dr. med. Dipl. Soz. Joachim Szecsenyi
Head
Dept. for General Practice and Health Services Research
University Hospital Heidelberg
Voßstr. 2, D-69115 Heidelberg
Phone: 06221 – 56 4745
Fax: 06221 – 56 1972
E-Mail: joachim.szecsenyi@med.uni-heidelberg.de

29. 10. 2013 – Version 1.0

Implementation/Intervention Coordinating Center

Dr. med. Werner Besier
Board Chairman
Genossenschaft Gesundheitsprojekt Mannheim e.G.
Liebfrauenstr. 21, 68259 Mannheim
Phone: 0621 -799600
Fax: 0621 – 7996029
E-Mail: praxis@dr-besier.de

Co-Investigators/Study Central Office

Dr. med. Kayvan Bozorgmehr (M.Sc.)
Research Fellow
Dept. for General Practice and Health Services Research
University Hospital Heidelberg
Voßstr. 2, 69115 Heidelberg
Phone: 06221 – 56-38581
Fax: 06221 – 56-1972
E-Mail: kayvan.bozorgmehr@med.uni-heidelberg.de

Dr. Dominik Ose, MPH
Research Fellow
Dept. for General Practice and Health Services Research
University Hospital Heidelberg
Voßstr. 2, 69115 Heidelberg
Phone: 06221 – 56-8012
Fax: 06221 – 56-1972
E-Mail: dominik.ose@med.uni-heidelberg.de

Dr. med. Tobias Freund
Research Fellow
Dept. for General Practice and Health Services Research
University Hospital Heidelberg
Voßstr. 2, 69115 Heidelberg
Phone: 06221 - 56- 8317
Fax: 06221 - 56-1972
E-Mail: tobias.freund@med.uni-heidelberg.de

Biostatistics

Johannes Krisam (M.Sc.)
Responsible Statistician
Institute for Medical Biometry and Informatics
Dept. for Medical Biometry
University Hospital Heidelberg
Im Neuenheimer Feld 305, 69120 Heidelberg
Phone: 06221 – 56-4369
Email: krisam@imbi.uni-heidelberg.de

Dr. Christian Stock (Dr.sc.hum., M.Sc., Dipl.Kfm.)
Supervising Statistician
Institute for Medical Biometry and Informatics
Dept. for Medical Biometry
University Hospital Heidelberg
Im Neuenheimer Feld 305, 69120 Heidelberg
Phone: 06221 – 56-5346
Email: stock@imbi.uni-heidelberg.de

Health Economic Evaluation

Prof. Dr. Hans Joachim Salize
Head
Research Group 'Health Services Research'
Central Institute of Mental Health
Phone: 0621- 1703-6401
Email: hans-joachim.salize@zi-mannheim.de

Christian Jacke (Dipl.-Sozialw.)
Research fellow
Research Group 'Health Services Research'
Central Institute of Mental Health
Phone: 0621/17036404
Email: christian.jacke@zi-mannheim.de

Monitoring/Study Central Office

Marion Kiel (Dipl. Oec. Troph.)
Clinical Monitor
Dept. for General Practice and Health Services Research
University Hospital Heidelberg
Voßstr. 2, 69115 Heidelberg
Phone: 06221 – 56-8072
Fax: 06221 – 56-1972
EMail: marion.kiel@med.uni-heidelberg.de

Annika Baldauf
Study Nurse
Dept. for General Practice and Health Services Research
University Hospital Heidelberg
Voßstr. 2, 69115 Heidelberg
Phone: 06221 – 56-8238
Fax: 06221 – 56-1972
EMail: annika.baldauf@med.uni-heidelberg.de

Data Management/Study Central Office

Markus Qreini
Dept. for General Practice and Health Services Research
University Hospital Heidelberg
Voßstr. 2, 69115 Heidelberg
Phone: 06221 – 56-8077
Fax: 06221 – 56-1972
EMail: markus.qreini@med.uni-heidelberg.de

Data Management/Intervention Coordinating Center

Beate Gerling
Geschäftsstelle
GGM Gesundheitsprojekt Mannheim e.G.
Liebfrauenstrasse 21
68259 Mannheim
Telefon: 0621-7900440
Fax: 0621-7900445
Email: gerling@ggm-gesundheitsprojekt.de

List of Acronyms

AMV	Dept. Of General Practice and Health Services Research, University Hospital Heidelberg
BMBF	Federal Ministry of Education and Research
DA	Dialogue assistant
FUPHV	Follow-up home visit
GGM	Genossenschaft Gesundheitsprojekt Mannheim e.G.
HCA	Health care assistant
HRQL	Health related quality of life
IQWIG	Institute for Quality and Efficiency in Health Care
IT	Information Technology
NCM	Net-case manager
PCP	Primary care practice
PCPnet	Primary care practice-net
PROs	Patient reported outcomes
PTC	Pathway to change
QALY	Quality Adjusted Life-Years
SDSCA	Summary of diabetes self-care activities measure
SDSCA-G	Summary of diabetes self-care activities measure, German version
SMBG	Self-monitoring of blood glucose
TAU	Treatment as Usual

Summary

Background:

The increasing prevalence of multiple, co-occurring chronic conditions places a burden for contemporary health systems. Care management interventions in the German healthcare system have been evaluated with promising results. However, further studies are necessary to explore the full potential of care management interventions for the improvement of outpatient diabetes care in the context of multi-morbidity in Germany.

Objective:

To assess the efficacy of a primary care practice net-based, IT-supported care management intervention with integrated telephone monitoring for patients with diabetes type II and multiple co-occurring chronic conditions.

Methods:

The study is designed as a prospective, 18-month, multi-center, investigator-blinded, two-armed, open-label, individual-level randomized parallel-group superiority trial (RCT). Study sites: 30 primary care practices. Participants: (i) 582 patients with diabetes mellitus type II and at least two severe chronic conditions and (ii) one informal care giver per patient. Data collection: Baseline (T0), primary endpoint after 9 months (T1), follow-up after 18 months (T2). Primary outcome: Change in diabetes related self-care behavior using a German version of the revised "Summary of diabetes self-care activities measure" (SDSCA-G) after 9 months. Secondary outcomes: Change in SDSCA-G at T2; mean change in each of the five dimension of self-care captured by the SDSCA-G; mean change in physician-reported glycosylated hemoglobin A1 (HbA1c %) at T1/T2; the cumulative incidence of physician-reported (severe) symptomatic hypoglycemia at T1/T2; mean change in patient-reported health related quality of life (HRQL) at T1/T2; mean change in patient-reported self-efficacy to cope with chronic conditions; differences in service utilization, costs of health care provision and financial burden of informal care between intervention and control group. Intervention: Is delivered by a trained health care assistant as add-on to usual care and consists of 3 main elements: (i) 3 home visits including structured assessment of medical and social needs; (ii) 24 structured telephone monitoring contacts; (iii) self-monitoring of blood glucose to create a 3-days-sugar-profile after T1 in 3-months intervals. All elements are IT-supported using the ICW Care Manager as intervention platform. Control: consists of usual care in the scope of the statutory health care system.

Analysis:

The confirmatory, primary analysis will be performed following the intention-to-treat (ITT) principle. The efficacy of the intervention will be quantified using two-level linear regression stratified by type of medical treatment (insulin vs. non-insulin therapy). The effect of the intervention on the primary outcome will be adjusted for baseline values in SDSCA-G. Secondary analyses (related to the secondary objectives) will be performed according to ITT principle as well, followed by a complete case analysis as sensitivity analysis to assess the robustness of effect estimates. To relate cost differential to outcome differential between intervention and control group the incremental cost effectiveness ratio will be calculated. We will apply bootstrap methods to model uncertainties in costs. Maximum willingness to pay thresholds and cost-acceptability curves will be determined. Sensitivity analyses will provide information on robustness of assumptions.

Index

List of Acronyms 3
Summary 4
1. Introduction 1
2. Study objectives 3
3. Methods 6
4. Study design 12
5. Inclusion criteria 13
6. Exclusion criteria 14
7. Randomization 14
8. Intervention/Study implementation 15
9. Recruitment of study centers, patients and patients' caregiving family members 20
10 Data collection 21
11 Statistics 22
12. Concomitant therapy 25
13. Safety laboratory 25
14. Termination criteria 25
15. Ethics and legal aspects 26
16. References 29
17. Unterschriften 34
18. Appendices 34

1. Introduction

The increasing prevalence of multiple, co-occurring chronic conditions places a burden for contemporary health systems (1, 2). Multi-morbidity is associated with worse health outcomes, more complex clinical management, and increased health care costs (3). Several suggestions are available to take up the challenge by re-organizing the delivery of chronic illness care (4, 5). Based on these concepts, care management interventions have been developed and evaluated focusing on patients with multiple chronic conditions (6). Care management refers to ‘a set of activities designed to assist patients and their support systems in managing medical conditions and related psychosocial problems more effectively, with the aim of improving patients’ health status and reducing the need for medical services’ (7). These interventions share four core elements (8): (a) comprehensive assessment of patients’ medical and non-medical needs and resources, (b) implementation and monitoring of individualized, evidence-based care plans, (c) coordination of services between providers of medical and social care, and (d) enhancement of self-management capabilities among patients and caregivers.

In the context of type II diabetes, the concept of “self-care” (a synonym for self-management) plays an increasingly important role in preventing complications and coping with consequences of the condition (9). Relevant interrelated self-care dimensions for patients with type II diabetes include adaptations to diet, exercise, self-monitoring of blood glucose (SMBG), foot care, medication adherence and smoking (10). Diabetes typically occurs with or has other chronic diseases as consequences. Discordant comorbidities, such as depression (11), constitute an additional challenge for patients with diabetes to effectively conduct self-care activities.

Evidence suggests that, at least in the short run, self-care behaviors can be modified through self-management interventions (12). However, it is important that interventions aimed at supporting patients to cope with diabetes and potentially co-occurring conditions are person-centered (not disease-centered), comprehensive, embedded in the process of primary care within the health care system and take into account and mobilize community resources (13), especially in the context of socially disadvantaged populations (14). Isolated interventions, that use didactic teaching alone (14) or rely on technological, computer-based approaches alone (15) have been shown to be less (14) or not effective to improve cognitive, behavioral or emotional outcomes (15).

Although structured disease management programs (DMP) are embedded in routine primary care for patients with type II diabetes in Germany(16), their ‘disease-centered’ design does not sufficiently take into account the challenge of comorbidity. For care management programs, however, positive effects on quality of care and patients’ quality of life has been reported (7). But still, the effect of care management interventions on healthcare utilization and costs remains heterogeneous (7). This finding is reinforced by a recent systematic review of complex interventions designed to improve outcomes in patients with multi-morbidity (including comorbidity) in primary care and community settings (17). The

review concludes that there is limited evidence on the care of patients with multi-morbidity, despite the prevalence of multi-morbidity and its impact on patients and healthcare systems. Included studies provided mixed results; partially due to the heterogeneity of included study populations as far as their chronic conditions are concerned. Research gaps were identified with respect to a paucity of economic analyses, the neglect of the impact of socioeconomic deprivation on multi-morbidity, and a small number of interventions with a clear definition of the 'multi-morbid' study population (17).

There are also specific research gaps related to care management among diabetes patients. Most interventions designed to improve the management of this population in primary care, outpatient or community settings have used biomedical surrogate parameters as primary outcome, and few have assessed the effects on patient reported outcomes (PROs) (13), such as self-care behavior, self-efficacy, attitudes, health service utilization, quality of life and/or psychological indicators (18). There are also few trials that report 'patient-important' outcomes related to death, morbidity (including hypoglycemic events) and quality of life. In a systematic review of 1057 ongoing and registered trials (in 2007), only 18% of trials reported such 'patient-important' outcomes (19).

The following features of interventions have been reported as promising to improve diabetes relevant outcomes (HbA1c, weight/BMI, lipids, blood pressure, health care utilization, physical activity), particularly among socially disadvantaged populations with diabetes (14): cultural tailoring, individualized assessment and reassessment, use of treatment algorithms by health care providers, focus on behavior-related tasks, use of feedback about patients' control of disease or performance, and high intensity interventions (> 10 contact times) delivered over a long duration (≥ 6 months).

Care management interventions in the German healthcare system have been evaluated with promising results for patients with osteoarthritis (20), depression (21), systolic heart failure (22), and index diseases such as diabetes mellitus, chronic obstructive lung disease, and chronic heart failure (6). However, further studies are necessary to explore the full potential of care management interventions to improve outpatient diabetes care in the context of multi-morbidity in Germany. Care management is particularly challenging to deliver for small primary care practices (PCPs). Practice-net based approaches might be a solution in this context, because they allow providing intensified care for patients from several practices, thus reducing the workload for smaller PCPs. These approaches in turn entail challenges regarding the flow of information between care managers, PCPs and other sectors in the delivery of relevant health care services according to individual need.

Based on an existing pilot project, we have developed a complex primary care practice-net (PCPnet) based, health-care-assistant (HCA) led, IT-supported care management intervention with integrated telephone-monitoring to improve diabetes care for patients with type II diabetes mellitus and multiple comorbidities. This study protocol documents the evaluation methods to be deployed during the 'evaluation stage' (23) of the complex intervention.

2. Study objectives

The primary objective of this study is to assess the efficacy of a PCPnet-based, HCA-led, IT-supported care management intervention with integrated telephone-monitoring for the improvement of self-care behavior among patients with type II diabetes mellitus and two or more comorbidities (see Chapter 8 and Figure 1 for a detailed description of the intervention).

2.1 Primary objective:

To determine, if there is a difference in the degree of change in diabetes related self-care behavior after 9 months (T1) of intensified care management (as add-on to usual care) compared to baseline (T0) between the intervention group (PTC) and the control group (TAU), which receives usual care only.

2.2 Secondary objectives

To assess:

1. if potential differences between both groups (PTC vs. TAU) in change of diabetes related self-care behavior compared to baseline (T0) can be sustained over an 18 months period (T2) with reduced intensity of care management and enhanced by self-monitoring of blood-glucose (whereas 'reduced intensity' refers to longer time-lags between telephone-monitoring contacts (Figure 1)).
2. if potential differences between both groups (PTC vs. TAU) in change of diabetes related self-care behavior after 9months (T1)/18 months (T2) compared to baseline (T0) are confounded by patients' behavior at base-line and/or other important factors reported in the literature (sex, age, marital status/domestic partnerships, social status, migration status, type of comorbidity, perceived quality of health care, alcohol abuse and diabetes related emotional distress).
3. the changes for each dimension of diabetes related self-care behavior (diet, exercise, self-monitoring of blood glucose (SMBG), foot care, and smoking behavior) at T1/T2 compared to baseline (T0)between both groups (PTC vs. TAU).
4. if there are differences at T1/T2 between both groups (PTC vs. TAU) in change of glycosylated hemoglobin A1 (HbA1c %) compared to baseline (T0) among the subgroup of patients with a baseline value of HbA1c > 7.5 % (adjusted for the base-line value of HbA1c)
5. if there are differences at T1/T2 between both groups (PTC vs. TAU) in the cumulative incidence of (severe) symptomatic hypoglycemia among those patients at risk of this event during the respective observation period
6. if there are differences at T1/T2 between both groups (PTC vs. TAU) in change of health related quality of life (HRQL) and self-efficacy compared to baseline.

7. if the care management intervention is equitable in the distribution of potential health gains (self-care behavior, HbA1c% and HRQL) at T1.
8. differences between the intervention- and the control-group related to service utilization, direct and indirect costs of health care provision as well as the financial burden of informal care provided by patients' relatives at the end of observation time.
9. the cost-effectiveness of the primary endpoint (change in self-care behavior) and the secondary endpoint (HRQL) at end of observation time (if the intervention is efficacious).

Primary outcome

The intervention primarily aims at improving "self-care" as a multi-dimensional construct consisting of the five dimensions diet, exercise, self-monitoring of blood glucose (SMBG), foot care, and smoking behavior. This primary outcome is a patient reported outcome (PRO) and will be operationalized by the German version of the revised „Summary of diabetes self-care activities measure" (10) (SDSCA). The English version of the revised SDSCA is a widely used, appropriate (24) valid and reliable tool with moderate test-retest-reliability (10) for capturing self-care behavior.

The German version (SDSCA-G) is based on a structured forward/backward translation of the original English instrument. The SDSCA-G will be deployed as part of paper-based questionnaire (see Appendix: Patientenfragebogen zur Erfassung der Selbstsorge) and consists of 11 items.

Scoring of items 1–10 (excluding smoking) uses days per week on a scale of 0–7 in which patients performed respective self-care tasks (24). For each group of items (e.g. diet) a mean number of days are calculated. The SDSCA scale is used as a continuous measure to determine if there are improvements compared to baseline at subsequent time points. As a result of strong ceiling effects, the 'medication' item is not included in the most recent recommended version [16].

No cut-off point has been determined to discriminate between adherence and non-adherence (24) and no minimal clinically relevant change in SDSCA 'scores' has yet been formulated (25). This means that, in order to determine a minimal clinically relevant change for this study, the research team needs to draw upon (i) published data on the degree of change in SDSCA values reported in interventions designed to improve self-management, and (ii) estimates about 'realistic' changes that the intervention can bring about.
The primary outcome used to assess the efficacy of the intervention is the difference in mean change in SDSCA-G 'score' of items 1-10 (excluding smoking) per patient compared to baseline (T0) after 9 months of intervention (T1) between both groups (PTC vs. TAU) determined on the basis of patients' self-reports.

Secondary outcomes

In light of the secondary objectives, the secondary outcomes of this study refer to:

1. The mean change in SDSCA-G 'score' of items 1-10 (excluding smoking) for each group (PTC vs. TAU) compared to baseline (T0) after 18 months of intervention (T2), to assess sustainability of effects with reduced intensity of care management and enhanced by self-monitoring of blood-glucose
2. The mean change in each of the five dimension of self-care captured by the SDSCA-G instrument compared to baseline (T0) for each group (PTC vs. TAU) at T1/T2 of intervention to assess the influence of the intervention on specific self-care behaviors
3. The mean change in physician-reported glycosylated hemoglobin A1 (HbA1c %) at T1/T2 compared to baseline among the subgroup of patients with a baseline value of HbA1c > 7.5 %. A 10 % improvement from the baseline value (25) after 9 months/18 months will be regarded as clinically relevant change.
4. The cumulative incidence of physician-reported (severe) symptomatic hypoglycemia at T1/T2 among those patients at risk of this event during the respective observation period. 'Symptomatic hypoglycemia' refers to hypoglycemia with blood glucose < 60 mg/dl, and 'severe symptomatic hypoglycemia' as those events of hypoglycemia, which necessitated external support. Patients are regarded 'at risk' of (severe) symptomatic hypoglycemia for the subsequent time period if they receive oral anti-diabetic medication and/or insulin therapy at T0 or T1.
5. The mean change in patient-reported HRQL, captured by the EuroQol instrument EQ-5D, at T1/T2 compared to baseline. EQ-5D is a 5-item, simple preference-based index measure which has frequently been applied in the measurement of HRQL in type 2 diabetes (including the United Kingdom Prospective Diabetes Study). The overall EQ-Index score (on a scale of 0 to 1) can be incorporated into Quality Adjusted Life-Years (QALY) for cost-utility analysis. A difference of 0.03 on overall EQ-Index score after 9 months/18 months will be considered to be clinically relevant (25).
6. The mean change in patient-reported self-efficacy, captured using the SES6-G instrument, a short and validated instrument with 6-items (26).
7. The equity-efficacy ratio calculated as the ratio between the change (compared to T0 at T1) in SDSCA-G 'score' of items 1-10 (excluding smoking), HbA1c% and EQ-Index among the lowest vs. highest socioeconomic group (including education, occupation, income and subjective social status) in both arms of the trial (PTC vs. TAU). This includes subgroup analyses for migrant populations.
8. Further secondary outcomes will be captured by the Client Socio-demographic and Service Receipt Inventory (CSSRI) (27, 28) and the financial family burden questionnaire. Both instruments have been adapted to the special needs of patients with type II diabetes and were

pretested before general application. CSSRI provides data on service utilization and refers to costs of health care provision and financial burden of informal care. Direct and indirect costs will be measured and analyzed from a societal perspective.

For an overview of all instruments used in this study to capture primary and secondary outcomes see Table 1. Physician reported secondary outcomes (HbA1c, events of hypoglycemia) are reported via a chart-review (for details see Chapter 10: Data collection).

3. Methods

3.1 General Aspects

The study is designed as a prospective, multicenter, two-armed, open-label, individual-level randomized controlled superiority trial (RCT). Since the main elements of the intervention are delivered by an NCM (for details see Chapter 8: Intervention/Study Implementation), it is feasible to have both intervention and control group based in each study center (i.e. in each PCP).

Eligible participants will be randomly selected from the overall patient population of 30 study centers (PCPs) and asked for their consent to take part in the study. Participating patients will be reported pseudonymized to the study central office (Dept. for General Practice and Health Services Research, AMV) and centrally randomized on an individual level by a clinical monitor, who acts as sole randomization authority. This person - not involved in care of the trial patients or analysis of data - will be responsible for allocation to control (treatment as usual, TAU) vs. intervention group (pathway to change, PTC) according to a pre-defined randomization schedule (for details see Chapter 7: Randomization). As illustrated in Figure 1, the intervention is additional to usual care. Blinding of either patients or practice teams is not possible due to the character of the intervention. The baseline (T0) assessment of primary and secondary outcomes will be conducted prior to allocation of patients, and after allocation at 9 months (primary endpoint: T1), and 18 months (follow-up: T2) (Figure 1).

Both intervention and control group receive usual care. Usual care consists of diabetes care in the scope of the DMP Diabetes. Care for high-prevalence chronic comorbidities such as chronic obstructive lung disease (COLD), heart failure (HF) and coronary heart disease (CHD) are also organized in the scope of DMPs on a voluntary basis in the statutory health insurance system (16). Care for other diseases (chronic or acute) is managed by PCP physicians individually based on personal experience, guidelines and/or patients' preferences.

Patients allocated to the PTC-group will be subject to the 18-month care management intervention as add-on to usual care. This complex care management intervention consists of three main elements (Figure 1):

(i) assessments by a net-case manager (NCM) including home visits,

(ii) regular telephone-monitoring contacts and

(iii) digital structured blood glucose monitoring (introduced after T1).

Within the first week after entry to the study, the NCM visits patients in the PTC group at home to conduct a standardized assessment of medical and non-medical problems (see Appendix: Studienhilfsmittel Formular „Erstkontakt Assessment“). The results of the assessment are reported to the physician in charge of caring for the patient via a designated dialogue assistant (DA) based at each PCP. DAs are HCAs tasked to facilitate communication between NCMs, PCPs and patients in both study arms.

In the second week, the patient visits his/her primary care physician in order to agree upon goals related to the identified problems. The goals will be documented and serve as the basis for the telephone-monitoring. A total of 24 telephone-monitoring sessions will occur according to the schedule presented in Figure 1 (every two weeks for a period of 6 months, once a month throughout the rest of the intervention period for patients in the PCT-group). The objective of the telephone-monitoring is to detect acute clinical needs and to provide support related to self-care goals and health care related issues. Follow-up home visits (FUPHV) will occur 6 months (FUPHV-1) and 12 months (FUPHV-2) after entry to the study for patients in the PTC-group (see Appendix: Studienhilfsmittel Formular „Folgekontakt Assessment“). Additionally, after 11 months, digital structured blood sugar testing is used for symptom-monitoring in a 3-months-interval (3 in total). The blood sugar profiles serve as the basis for the coaching of patients by the NCMs in the scope of the telephone-monitoring. NCMs support patients in the PTC-group to locate and access additional services within the health system or the broader community according to patients' individual preferences and needs.

A preceding, unpublished scoping review (29) conducted by the investigators identified relevant determinants of diabetes self-care to be considered as potential confounders in the context of this RCT. These include general demographical aspects (sex, age, marital status/domestic partnerships), the individual social status (education, income, occupation), cultural factors potentially related to the migration status, type of comorbidity (e.g. discordant ones such as depression (11, 30) and/or arthrosis), type of diabetes therapy (insulin vs. oral medication), perceived quality of health care, alcohol abuse and emotional distress. The potential confounders will be captured at T0-T2 as PROs. We further account for the type of medical therapy (insulin vs. oral medication/no medication) during the randomization process through stratified randomization (see Chapter 7: Randomization). For a full list of instruments and variables used in this study see Table 1 and Table 2.

Study findings will be reported according to the CONSORT 2010 checklist with extensions for PROs in RCTs (31).

Table 1 Overview of instruments, constructs and number of items

Instrument	Acronym	Construct	Items	Source
Summary of diabetes self-care activities measure – German version	SDSCA-G	Diabetes self-care	11	Toobert et al. (10)/own translation
"Kurzfragebogen Selbstsorge Diabetes"	Prac-Man Selbstsorge	Diabetes self-care	7	Freund et al. (6)
Chronic disease related self-efficacy (SES6G)	SES-6G	Self-efficacy	6	Freund et al. (26)
The Patient Assessment of Care for Chronic Conditions	PACIC short	Perceived actions or quality of care	11	Götz et al. (32)
Problem Areas in Diabetes (Short)	PAID Short	Emotional Dis-stress	5	ÄZQ (33)
WHO (Five) – Questionnaire	WHO 5	Depression	5	Psychiatric Research Unit, WHO Collaborating Center for Mental Health, Frederiksborg General Hospital (34)
EQ-5D (EuroQol Group)	EQ-5D	HRQL	5	Rabin et al. (35)
Audit-C-Questionnaire	Audit C	Alcohol use	3	Suchtforschungsverbund Baden-Württemberg (36)
Client Socio-demographic and Service Receipt Inventory	CSSRI	Health care utilization and costs	97	Roick et al./Chrisholm et al. (27, 28)
Financial family burden	FFB	Costs of informal care provided by a designated family member	55	Health services research group, Central Institute of Mental Health
**Total no. of instrument items in patient questionnaire**			150	
**Total no. of items in questionnaire targeting patients' caregiving family member**			55	

Table 2: Overview of variables and number of items in patient reported questionnaire

Variable	Items	Source
HbA1c %	1	-
Insulin therapy	1	-
Comorbidities	1	-
Participation in Disease Management Programs	1	-
Migration status	7	Schenk et al. (37)
Sex	1	Sociodemographic Standards of the Federal Department of Statistics (partially adapted) (38)
Age	1	
Zip code	1	
Housing	1	
Partnerships	2	
Education	2	
Occupation	3	
Equalized household income	3	
Subjective social status	1	German General Social Survey (ALLBUS) (39)
**Total no. of items**	26	

3.2 (Therapeutic) Effects

The above-mentioned elements (Figure 1) are hypothesized to be the 'active ingredients' (23) of the complex intervention. They are hypothesized to exert their effect in the following way:

NCM-led needs assessment including home visits:

Individualized assessment (including repeated reassessment) of medical and non-medical needs by non-physician health workers has been reported as important element in attempts to improve diabetes related outcomes such as HbA1c, weight/BMI, lipids, blood pressure, health care utilization, physical activity (14). Further elements leading to improved outcomes are the use of decision algorithms by health care providers and a focus on behavior-related tasks (14), both of which are incorporated in the IT-supported assessment tool deployed in this intervention. The focus on both medical and non-medical needs ensures that the intervention is person-centered (not disease centered) and thus coherent with the evidence on how to improve diabetes care (13). The assessment of patients' needs in their own environment is a reach-out element which ensures that needs are assessed comprehensively, taking into account the social environment of the patients. The assessment is embedded in the process of primary care within the health care system and mobilizes community resources in order to meet patients' identified medical and non-medical needs. This approach aims to reduce barriers for patients in using support systems and medical/non-medical services based in their local communities, in order to improve management of the disease. There is evidence that the combination of these features is effective in improving diabetes care (13).

However, due to the paucity of research into the effects of care management interventions on PROs in general, the positive effects on the primary and secondary outcomes used in this study can only be hypothesized.

NCM-led structured telephone monitoring:

The NCMs will deliver to each patient in the PTC-group a minimum of 24 structured, telephone monitoring contacts throughout the 18-month period (Figure 1). Evidence suggests that such high intensity interventions (>10 contact times) delivered over a long duration (≥6 months) are necessary in order to improve and sustain any change in self-care behavior, especially in "hard to reach" populations (14). Application of this element in care management interventions has been shown to be effective in early diagnosis and treatment of acute complications and problems, thereby reducing avoidable hospitalizations among chronically ill patients in the German health care context (6). The monitoring is also used as feedback instrument related to patients' control of disease or performance, which is also important to reinforce positive behavior change.

Digital structured self-monitoring of blood glucose:

Digital structured self-monitoring of blood glucose (SMBG) will be deployed for the PTC-group after T1 in a 3-month interval using Roche Diagnostics' Accu Chek® Smart Pix Device and ACCU-Chek® 360°.

The results are used to monitor trends in glycemic control and progress towards improved self-care behavior, and will be used as feedback tool towards patients in terms of bio-feedback.

SMBG is an essential self-care element among patients with insulin treated type 2 diabetes. However, for non-insulin treated diabetic patients a synthesis of the existing evidence published by the German Institute for Quality and Efficiency in Health Care (IQWIG) concludes that there is no evidence that SMBG improves diabetes related outcomes (40). However, as noted by the IQWIG report, these conclusions need to be interpreted in light of scarce data, especially in terms of potential effects on the incidence of severe hypoglycemia in non-insulin treated patient populations (40). A recent systematic review by the Cochrane Collaboration including 12 RCTs (duration range: 6-12 months) concluded that "[..] when diabetes duration is over one year, the overall effect of self-monitoring of blood glucose on glycemic control in patients with type 2 diabetes who are not using insulin is small up to six months after initiation and subsides after 12 months. Furthermore, based on a best-evidence synthesis, there is no evidence that SMBG affects patient satisfaction, general wellbeing or general health-related quality of life. More research is needed to explore the psychological impact of SMBG and its impact on diabetes specific quality of life and well-being, as well as the impact of SMBG on hypoglycemia and diabetic complications." (41)

The use of self-monitoring for non-insulin treated people has been controversial because estimates of benefit in reducing levels of HbA1c vary between systematic reviews (42). Meta-analysis using individual patient data did also not provide convincing evidence to support the routine use of self-monitoring of blood glucose in people with non-insulin treated type 2 diabetes (42). Thus, better evidence that self-monitoring can provide feedback on treatment and behavior are needed (42).

The 'scarce-data-argument' and the potential effects on the incidence of severe hypoglycemia are the main rationale for including this element in the intervention for non-insulin treated patients with type 2 diabetes after T1 in order to add to the existing evidence base in the scope of a secondary, descriptive analysis.

3.3 Risks and (unintended) effects

3.3.1 Risks and (unintended) effects of participation in the study for patients in both groups (PTC and TAU)

In the course of the study, both groups (PTC vs. TAU) undergo clinical assessments at T0-T2 and fill out questionnaires related to their self-care behavior. The clinical assessments mainly contain chart-reviews by PCP physicians, routine measurement of weight, blood pressure and history taking

regarding new diagnoses and/or medications. Unintended effects mainly refer to time issues and potentially cost issues linked to these additional doctor visits. However, given that the intervention takes place over a period of 18 months, the additional visits appear to be justifiable in terms of both time and costs. Filling out the questionnaires related to patients' self-care behavior can trigger positive as well as negative consequences: On the one hand, it is possible that patients become increasingly aware of their daily behavior in the context of their diabetes, and use this awareness as a resource to adapt the behavior accordingly. On the other hand, it is also possible that patients' awareness about individual weaknesses related to self-care issues results in desperation and blaming of ones 'self'. This is an inherent danger in the concept of 'self-care' which gains particular relevance in light of the combination of patients' self-reports on self-care behavior with the financial family burden (FFB) questionnaire. Since the FFB attempts to capture the burden of costs of informal care provided by a designated family member of the patient, feelings of guilt and blaming of ones 'self' about the consequences of the disease could be perpetuated.

To hamper the outlined potential unintended effects, all PCP physicians will be informed about the possibility of these potential effects (in the information leaflet for physicians). In order to qualify as study center, PCP physicians must give their written consent to offer extra-consultations to all participating patients in case they want to discuss or report any unintended effects. Furthermore, participating patients are encouraged to contact the principal investigator at any time throughout the course of the study to discuss potential risks and find solutions to potential problems.

3.3.2 Risks and (unintended) effects of participation in the study for patients in the intervention group (PTC)

In addition to the above, patients in the intervention group are exposed to a complex intervention (Figure 1), consisting of (in total) at least one extra face-to-face contact with the PCP physician to agree upon goals, 3 home visits and clinical assessments by a NCM, 24 telephone-monitoring sessions performed by a NCM and 3 events of digital structured blood glucose testing using Roche Diagnostics' Accu Chek® Smart Pix Device and ACCU-Chek® 360°.

The extra contact to the PCP physician, home visits and telephone-monitoring sessions are all amenable to the previously mentioned unintended effects regarding time and cost.

During the home visits, NCMs gain insights into patients' private environments. Patients might feel uncomfortable with this situation, particularly if they are socially disadvantaged and live in deprived neighborhoods or conditions. During the training of NCMs, the GGM will place particular emphasis on confidentiality, and on effective strategies to handle such situations.

Further risks and unintended effects for patients in the PTC group after T1 might be involved in the digital structured blood glucose testing using Roche Diagnostics' Accu Chek® Smart Pix Device and ACCU-Chek® 360°. In general, there is a paucity of research reporting data about risks and unintended

effects of SMBG among patients with type II diabetes who do not use insulin (40). In light of scarce data, a report of the Institute for Quality and Efficiency in Health Care (IQWIG) concludes that – for this group of patients – no clinically relevant side effects have been reported as far as HRQL is concerned, and no statistically significant difference as far as patient satisfaction or 'other unintended effects' are concerned (40). However, qualitative studies often report unease, uncomfort or pain related to SMBG. NCMs will be trained to enquire about these side effects and discuss appropriate strategies with the PCP physician, should a patient feel considerably impaired by this element of the intervention. In this context, the patient decides whether or not he/she feels 'considerably impaired'.

3.3.3 Risks and (unintended) effects of participation in the study for patients' designated relatives in the intervention group (PTC)

All patients in the intervention group (PTC) are asked (at T0-T2) to hand a questionnaire to one designated family member, who – in the patients' view – cares most for them as far as their diabetes is concerned. This financial family burden (FFB) questionnaire captures the costs of informal care provided by a family member. Filling out the FFB questionnaire can trigger positive or negative effects: On the one hand, the patients' family member might become aware of the cost (including financial and time resources) involved in caring for his/her relative with type II diabetes and use this awareness as resource. On the other hand, the awareness of the potential burden of informal care might, in the worst case, lead to victim-blaming towards the patient with type II diabetes.

To hamper the outlined potential unintended effects, all PCP physicians will be informed about the possibility of these potential effects (in the information leaflet for physicians). Additionally, all patients will be informed about the contents of the FFB questionnaire, so that they can decide whether or not they hand on the questionnaire to their 'caring' family member. In order to qualify as study center, PCP physicians must give their written consent to offer extra-consultations to all participating patients in case they want to discuss or report any unintended effects in this context. Unintended effects will be captured by the physician by means of a pre-determined reporting form if reported by a patient (see Appendix: Studienhilfsmittel "Meldebogen Unerwünschte Ereignisse"). Furthermore, participating patients are encouraged to contact the principal investigator at any time throughout the course of the study to discuss potential risks and find solutions to potential problems linked to the FFB questionnaire.

4. Study design

The study is designed as a prospective, 18-month, multi-center, two-armed, open-label, individual-level randomized parallel-group superiority trial (RCT). The control group receives treatment as usual (TAU), the intervention group (PTC) receives TAU **and** a complex care management intervention. Due to the character of the applied intervention, blinding of patients or practice teams is not possible.

5. Inclusion criteria

5.1 Inclusion criteria for primary care practices (PCPs)

To function as a study center, PCP physicians must fulfill the following criteria: Specialized in General Practice, Internal Medicine or Practical Physician (praktischer Arzt) and function as "Primary care physician" (Hausarzt) according to German regulations. Both single and group practices (Gemeinschaftspraxen) are eligible to participate. Inclusion criteria for PCP will be captured by a brief, personalized questionnaire (see Appendix: Studienhilfsmittel "Soziodemografie_Ärzte_v1.0_22.10.2013").

5.2 Inclusion criteria for patients

To be eligible for participation in the study, patients have to be:

- Aged 18 years or above,
- Diagnosed with type 2 diabetes mellitus (ICD 10: E11-E14),
- Enrolled in the Disease-Management-Program Diabetes mellitus type 2 (DMP Diabetes), and
- Diagnosed with at least two severe chronic comorbidities („schwerwiegende chronische Erkrankung“) according to the definition enshrined in § 62 SGB V (43). These include, but are not limited to, atherosclerosis (ICD 10: I70), chronic coronary heart disease (ICD 10: I25), chronic obstructive lung disease (ICD 10: J44), asthma (ICD 10: J45), cerebrovascular diseases (ICD 10: I60-I69), Depression (ICD 10: F32-F33), heart failure (ICD 10: I50), Parkinson (ICD 10: G20) and/or chronic pain (ICD 10: R52).

Additionally, written informed consent is a prerequisite for participation in the study.

5.3 Inclusion criteria for a patient's caregiving family member

One of the secondary research objectives is to assess costs of informal care in both groups (PTC vs TAU). This will be collected by the FFB questionnaire (see Appendix: Angehörigenfragebogen), which addresses the family member who 'cares most' for the respective patient with type II diabetes. A caregiving family member is defined as

- The family member who (from the patients' perspective) spends the largest amount of time with informal care (e.g. spouse, partner, father, mother, son, daughter, others)
- and lives in the same household.

It is important to note that it is up to the individual patient to identify, from their social relationships, the family member 'who cares most'. As such, this construct explicitly considers patients' views and does not apply to an external definition. Only one family member per patient will be recruited. It is important to note that the patient decides whether or not to pass on the questionnaire along with the informed consent and information material to his/her selected informal caregiver.

6. Exclusion criteria

6.1 Exclusion criteria for primary care practices (PCPs) and patients' caregiving family members

PCPs and patients' caregiving family members who do not fulfill the inclusion criteria will be excluded.

6.2 Exclusion criteria for patients

Patients who do not fulfill the inclusion criteria will be excluded. Additional exclusion criteria are patients with:

- Severe acute psychiatric disorders, e.g. Schizophrenia, schizotypal and delusional disorders (ICD 10: F20-F29)
- Dementia (ICD 10: F00-F03)
- Mental and behavioral disorders due to psychoactive substance use (ICD 10: F11-F16; F18; F19) (except for alcohol (ICD 10: F10) and tobacco use (ICD 10: F17))
- Malignant neoplasms (ICD 10: C00-C97) and/or current chemotherapy or radiotherapy
- Transplanted organ and tissue status (ICD 10: Z94)
- Care involving dialysis (ICD 10: Z49)
- Insurmountable language and communication problems
- Emergency cases

7. Randomization

Patients will be randomly allocated to case management (PTC) or usual care (TAU) at individual-level in the ratio of 1:1 stratified by

(i) type of medical treatment (insulin vs. oral medication/no medication) of their index disease (type 2 diabetes) and

(ii) study center (PCP).

Type of medical treatment has been identified as the strongest predictor of diabetes self-care in the preceding (unpublished) scoping review, and is thus captured by a physician reported questionnaire to serve as validated stratification criteria. We will hence perform stratified block randomization with variable block lengths to ensure study groups of approximately equal size. The block sizes will not be disclosed, to ensure concealment. Instead, this specific information will be provided in a separate document with restricted access only to the randomization authority. The randomization procedure is closely linked to the data collection procedure (see Chapter 10) in so far as that it is performed centrally using the ICW Care Manager as platform.

All eligible patients participating in the study will be registered in the software during a three-month wash-in phase, along with the physician-reported information about eligibility criteria (see Chapters 5 & 6) and type of medical therapy. The ICW Care Manager contains virtual folders for the two groups (PTC vs. TAU) within participating primary care practices.

The randomization authority (a designated person not involved in care of the trial patients and analysis of data) will access the database of registered patients in a weekly interval to monitor the recruitment process. Recruited and registered patients who fulfill the inclusion criteria will be centrally randomized (stratified by type of medical therapy and PCP) to PTC vs. TAU using a computerized random number generator to create randomization lists for each stratum. The allocation will be documented separately in an Excel file with restricted access except for the randomization authority.

Patients' allocation to either intervention or control group will remain concealed until data collection related to primary and secondary outcomes at baseline (T0) has been completed. After baseline data collection has been completed, PCPs will be informed about the allocation of "their" patients (identified through their unique study-ID) with an official letter from the intervention coordinating center (GGM), who in turn receives the information from the study coordinating center (AMV).

8. Intervention/Study implementation

The intervention is a complex case management intervention (23) aimed at improving diabetes self-care behavior among patients with type 2 diabetes and multiple comorbidities. The development of the multifaceted intervention (Figure 1) is based on:

(i) experiences of a 12-month pilot study of the GGM (GEDIMA) including 171 patients at baseline
(ii) focus groups with PCP physicians and specialist care providers involved in diabetes care, and
(iii) the active engagement of local patient self-help groups in formulating assessment contents and identifying community resources.

Detailed evaluation of the pilot study is still ongoing and will be completed end of October 2013 (pers. communication W. Besier/GGM). Preliminary results show a high acceptance among patients (N=171 at baseline, N=151 after 12 months, drop-out rate: 11.7%). The feasibility of the HCA-led, PCPnet-based care management intervention has been sufficiently tested among the 30 PCPs participating in the current study.

The complex intervention is delivered as add-on to usual care and consists of 3 main elements:

Assessment

At the beginning, patients in the PTC-group receive an IT-based assessment (see Appendix: Studienhilfsmittel Formulare "Erstkontakt Assessment"/"Folgekontakt Assessment") of medical and non-medical needs and resources using a structured protocol by a PCP-net based HCA who functions as net-case manager (NCM).

The NCM is authorized to the ICW Care Manager with a unique password and username. At the beginning, the NCM has only access to pseudonymized data of those recruited patients in the intervention arm, which are based at the PCPs for which she is in charge. The NCM contacts the PCPs in order to de-pseudonymize 'her' patients and to deliver the assessment.

Contents of the assessment are: demographics (age, sex, insurance status, living environment, education, migration status/cultural background), utilization of existing health and social services related to individual need (participation in DMPs beyond DMP Diabetes, hospitalization in the last 12 months, social services (Sozialdienst), home nursing services (Pflegedienst), medical aids, physiotherapy, podiatrist, psychotherapy, selected symptoms/clinical manifestations (thirst, lack of appetite, nausea, vomiting, dyspnea, fatigue, wounds, disorientation), biomedical parameters (blood pressure, weight, height, blood sugar), SMBG, participation in diabetes education programs, problems in organization of care and social situation, tripping hazards and social resources (external care givers, family, neighborhood).

Additionally, the NCM performs the following standardized, clinical assessments: mini mental status test (MMST), activities of daily living (I-ADL), tandem stand. Depression (WHO5), mobility/physical activity, alcohol use (AUDIT-C) and tobacco use (pack years) are assessed to identify entry points for individual support. The IT-platform provides the NCM with (physician reported) background information related to diagnoses, medication, HbA1c, proteinuria and potentially existing patient decrees (Patientenverfügung, Vorsorgevollmacht, Betreuungsverfügung).

The results of the initial assessment will be reported to a dialogue assistant based at the PCP, who in turn reports to the caring PCP in charge of the patient. The assessment results serve as the basis to set common goals between patient and PCP based on individual need.

Assessment results will be digitally stored by the software on a secured server (see Chapter 15) to inform the NCM during follow-up (FUP) home visits and during the telephone-monitoring. FUP home visits will occur 6 months (FUP1) and 12 months (FUP2) after entry to the study for patients in the PTC-group (Figure 1).

Symptom and Needs Monitoring

NCMs will deliver a regular telephone-monitoring using a structured monitoring list (see Appendix X). Content and frequency of the monitoring are fixed, but additional items may be determined by the PCP and the NCM. The NCM will deliver (at least) a total of 24 telephone-monitoring sessions according to a fixed schedule (2-weekly for a period of 6 months, monthly for the rest of the intervention period for patients in the PCT-group (Figure 1). Monitoring items contain new diagnoses, dyspnea, incidence/frequency/severity of hypoglycemia, blood pressure, weight, fatigue, medical/social problems and follow-ups on previously mentioned problems and hospitalizations since the last contact. The monitoring list (see Appendix: Studienhilfsmittel "Checkliste Telefonmonitoring") has been developed based on experiences of GGM in prior studies (GEDIMA). Item responses include 'red flags' according to the urgency of the symptoms and signs presented by the patient. These responses will guide the NCM-Patient-PCP interaction. 'Red flag' answers require immediate contact to the PCP to discuss and undertake further steps.

Digital structured blood glucose testing

Additionally, digital structured blood sugar testing (using Roche Diagnostics' Accu Chek® Smart Pix Device and ACCU-Chek® 360°) is applied after T1 for symptom monitoring in 3-monthly interval (in total 3 times) (Figure 1). The NCM collects the individual blood sugar profiles, transfers them into the ICW Care Manager and reports the profiles to the PCP in charge of respective patients. The blood sugar profiles serve as basis for the telephone coaching.

Composition and training of case management teams

The care management teams consist of 11 NCMs, 30 PCP physicians and 30 so called dialogue assistants (DA) based at each PCP. Each NCM cares for about 26 patients from about 3 PCPs.

N=3 NCMs have previously participated in care management interventions related to diabetes and CHD (GEDIMA and KHK-ProMA), N=2 have previously participated in care management interventions related to CHD alone (KHK-ProMA), N=6 have no experience with care management programs.

N=1 NCM is a native Turkish-speaker specialized in the provision of diabetes education. This NCM will be responsible to deliver the care management intervention to patients with a Turkish background in case of language barriers or cultural preferences. We thereby aim to address the special needs of this social group, which constitutes about 22% of the population (on December 30, 2012) in the city where the majority of study centers (PCPs) will be based (i.e. Mannheim, Germany). Potential language barriers to data collection will be accounted for by means of a Turkish version of the patient questionnaire.

Prior to the beginning of the intervention, all NCMs will be trained according to a team-based training curriculum. The curriculum was developed based on experiences from the preceding pilot study (GEDIMA) and inputs from representatives of local patient self-help groups. Completion of a 32 hours team-based training course is mandatory for participating HCAs to function as NCM.

Control

In the control group (TAU), practice teams will continue to provide standard care in the context of the PC-centered care contract. This involves gate keeping for enrolled patients as well as regular training in evidence-based guidelines through structured pharmacotherapy feedback in peer review groups [39]. As population-based disease management programs (DMPs) for diabetes and other selected chronic diseases are part of routine care in Germany, patients may voluntarily participate in these disease specific care programs. German DMPs consist of regular follow up visits up to every three months. They include clinical examination, laboratory tests (e.g. HbA1c tests), patient education, and referral to specialists (opthalmologists, cardiologists, nephrologists, neurologists) on a regular basis and according to acute needs (16). However, essential elements of care management interventions like individualized assessment, care planning including non-medical aspects, home visits, and frequent (symptom) monitoring are not routinely part of DMPs (6).

Fidelity to the study protocol and variability between sites and care management teams

The intervention seeks to ensure fidelity to the study protocol as far as adherence to the elements of the intervention and the timeline (see Figure 1) are concerned. The IT-supported delivery of the intervention allows assessing fidelity to the main intervention elements for all participants in the intervention group. However, the intervention is explicitly designed to allow adaptations to local contexts, individual needs, preferences and cultural backgrounds as far as the consequences of structured assessment and telephone monitoring are concerned. Adaptations to local contexts and individual preferences (e.g. differential use in community resources and services) will be documented in the ICW Care Manager by the NCM during her routine contacts.

Figure 1 illustrates the elements and the timeline of the intervention.

Figure 1: Graphical illustration of elements of the complex intervention and the study timeline

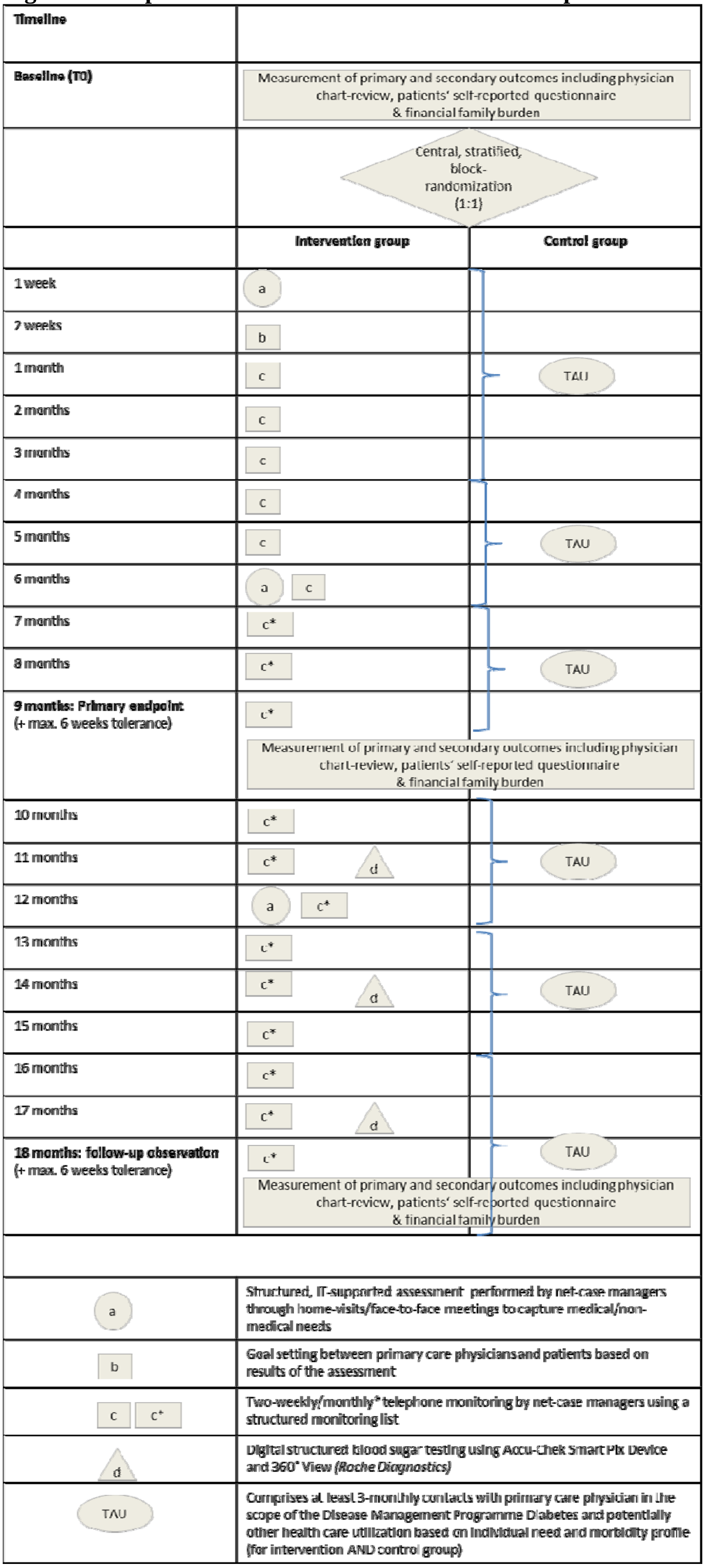

Adapted from: Perera et al. (2007) (44)

9. Recruitment of study centers, patients and patients' caregiving family members

9.1 Study centers

The multicenter RCT will be conducted in 30 PCPs. PCPs who formerly participated in a pilot study (GEDIMA) of the GGM will be invited by an official letter from the study central office to participate in the study and need to give their written consent for participation.

9.2 Patients

From the study central office (via the intervention coordination center GGM), the 30 PCPs receive a list with inclusion and exclusion criteria for patients (see above) along with a screening list with random numbers. PCPs are asked to create a list of all potentially eligible patients registered in their practice software based on the inclusion and exclusion criteria.

In a next step, PCPs select 40 patients from the list according to the sequence indicated by the random numbers. Randomly selected patients will be contacted and asked for participation in the study by the PCP physician. Patients not willing to participate will be asked to give a reason for their decision (on a voluntary basis). The number of patients screened, asked, asked and not included as well as the reasons for inclusion, exclusion or non-participation will be documented by the PCP physician and reported to the study central office.

The procedure will be repeated until a total of 20 patients per PCP are recruited.

Patients willing to participate will be informed by the PCP physician about aims, content, privacy issues and risks related to the study. Patients giving their consent to participate in the study will undergo clinical examination and will receive the patient questionnaires (see Appendix) which capture the PROs.

9.3 Patients' 'caring' family member

Patients giving their consent to participate in the study will be asked to hand over (on a voluntary basis) an information leaflet, an informed consent form and a questionnaire in a closed-envelope to their family member who 'cares most' for them. The participation of the 'chosen' family member is completely voluntarily, and independent from the participation of the patient who 'chooses' the family member (for details see Chapter 10: Data collection). Data collected from the patients' family member will be pseudonymized in a way that allows linking the informal care burden with the disease severity of respective patients.

9.4 Financial incentives

Participating PCPs receive a compensation per recruited patient and completed assessment at baseline (To: 25,- €), endpoint (T1: 30,- €) and follow-up (T2: 25,- €) respectively (in total 80,- €). DAs receive 20 Euros per patient per PCP for coordination and communication tasks. NCMs enter a minor employment contract with the GGM (on a 400,- Euro basis) in order to ensure compensation for the delivery of the intensive care management intervention. Participating patients and their respective family members receive no compensation.

10 Data collection

After obtaining written informed consent, patients will be registered in the study central office of the Department of General Practice and Health Services Research Heidelberg (AMV) which is responsible for administration, coordination, data management and monitoring (includes database-set up and validation, data entry, coding, and query management).

The intervention coordinating center of the "Genossenschaft Gesundheitsprojekt Mannheim e.V." (GGM) will carry joint responsibility for administration, validation and data entry.
Patients will be asked by the recruiting physician to fill in a pseudonymized paper-based questionnaire and hand them back to the recruiting physician in a closed envelope. This questionnaire (see Appendix: Patientenfragebogen) captures the patient reported outcomes (PROs) analyzed in this study.

PCPs will document additional data from patients' chart in a pseudonymized paper-based questionnaire (including inclusion/exclusion criteria, diagnoses, medication, and hospitalization/hypoglycemia prior to recruitment) and assess the clinical status of the patient (e.g. blood pressure, blood glucose, latest HbA1c value). The pseudonymized physician questionnaire will not contain any data that allows identification of patients' identity. The patient questionnaire and physician reported chart-review will be performed at baseline (To), 9 months (T1), and 18 months (T2) follow-up.

In addition, patients will be asked to hand over the FFB questionnaire (in a closed envelope along with additional background information) to their relative who 'cares most' for them regarding their diabetes, in order to capture the cost of informal care for the health economic evaluation. This questionnaire will be pseudonymized in a way that allows a linkage to the study ID of respective patients.

The collected data is directed to the study central office (AMV) via the following channels:

1. Physician reported chart-review and patient questionnaires will be sent by the PCPs in paper-based format to the study coordination center (GGM) via mail. Here, an authorized employee of GGM

enters the chart-review data into the ICW Care Manager and thereby registers participating patients with their study ID. All data entered into the ICW Manager will be transferred (via internet) to the Center for Information- and Medical Technologies (Zentrum für Informations- und Medizintechnologie, ZIM) and stored on a secured server (see Chapter 15: Ethics and legal aspects). Authorized clinical monitors based at the AMV can access the stored data in 'real time' in order to check conformity with inclusion criteria and in order to monitor the progress of the recruitment process. Validation of entered data occurs via built-in 'checks' by the ICW Care Manager and by clinical monitors.

2. The paper-based patient questionnaires containing the chart reviews and PROs will be stored at the GGM office and picked up by an employee of the study central office (AMV) in regular intervals.

3. The FFB questionnaire designed for patients' relatives will be sent directly to the AMV by patients' relatives using enclosed, postpaid envelopes. The written consent forms for patients' relatives will be sent to the GGM.

Patient questionnaires and FFB questionnaires will be scanned and digitally stored on a secured server of the AMV.

10.1 Monitoring

The following risk adapted strategy will be deployed to ensure adherence to the study protocol as far as data collection and legal/ethical aspects are concerned. Authorized employees of the study central office (AMV) will conduct practice visits at 10% of PCPs randomly selected from the pool of participating PCPs during the first 3 months of the intervention. The monitoring will include:

(i) an inspection of informed consents and patients existence of all patients recruited by the PCP,
(ii) a review of patient charts of 10% of patients recruited by these PCP's in order to check conformity among documented items (see Appendix: Arztfragebogen für Ersterhebung) and source data.

A detailed monitoring manual will be developed by the study central office prior to the beginning of the intervention. Depending on the results of these visits, further quality assurance measures will be defined by the study central office (AMV) and the intervention coordinating center (GGM).

11 Statistics

A detailed description of the statistical methods of this study will be provided in a Statistical Analysis Plan (SAP). Data analysis will be done blinded to treatment arm allocation (i.e. the treatments will be identified as 1 and 2 until analysis is complete). The primary analysis will be based on the 9-month follow-up data (T1).

11.1 Populations for analysis

The Intention-to-treat (ITT) population will consist of all randomized patients. Following the ITT principle, patients will be analyzed in the treatment arms to which they were originally randomized, regardless of whether they refused or discontinued treatment, or whether other protocol deviations are known.

The Per-protocol (PP) population will consist of those ITT patients with no major protocol violations. The criteria for the exclusion of patients from the PP population will be determined by the study team at the latest before database lock.

11.2 Statistical hypotheses, methods and analyses

The primary objective of this study is to determine the effectiveness of a complex intervention compared to usual care. The primary efficacy endpoint is the change in SDSCA-G score from baseline (T_0) to 9 months after baseline (T_1), i.e. the difference SDSCA-G T_1–T_0. The study objective will be statistically formulated as a test of the null hypothesis H_0: $\mu_1 = \mu_2$ (the mean difference SDSCA-G T_1–T_0 is equal in the two groups) against the alternative hypothesis H_1: $\mu_1 \neq \mu_2$ (the mean differences SDSCA-G T_1–T_0 are different in the two groups). The null hypothesis will be tested at the two-sided significance level of $\alpha=0.05$.

The primary efficacy analysis will use a multilevel regression approach with patients at level one and NCMs at level two. The primary model will include treatment group as fixed factor, NCMs as random factor with the baseline value of SDSCA-G and type of medical treatment as covariates. The results will be presented as the mean between-group difference in SDSCA-G T_1–T_0 with the corresponding 95% confidence interval. The associated Cohen's effect size d will be calculated. To support the primary analysis, all potentially relevant baseline characteristics will be added as covariates to the model in sensitivity analyses. A further sensitivity analysis of the primary endpoint will include an unadjusted two-sample t-test on change in SDSCA-G from baseline to 9 months after baseline. Results from these sensitivity analyses will serve to explain and interpret the results of the primary analysis.

The primary analysis will be performed adhering to the intention-to-treat principle. An additional sensitivity analysis will be conducted on a per-protocol analysis set.

The statistical analyses of the secondary endpoints numbered 1-6 (see pages 4-5) will use the same multilevel approach as the primary analysis. According to the scaling of the considered endpoint, a linear or logistic two-level regression model will be fitted. The results will be presented in an analogous manner to the primary analysis. All statistical tests will be two-sided at the significance level of $\alpha=0.05$.

The equity-efficacy ratio (45) (and respective 95 % confidence intervals) will be calculated as the ratio of effects at T1 on health outcomes (change in SDSCA score; HbA1c%; HRQL) between the highest and the lowest socioeconomic group (related education/income/subjective social status) for both groups (PTC vs. TAU) compared to T0. Additional consideration of social group sizes will be ensured by calculating the concentration index (PTC vs TAU) and plotting concentration curves at T0 and T1 respectively.

Subgroup analysis for nominal social groups will be performed by means of interaction terms for migration status (Turkish migrants, non-Turkish migrants, participants without migration background) to be included in the regression equations for respective health outcomes (change in SDSCA score; HbA1c%; HRQL) at T. If the tests of subgroup interaction effects are significant below the 0.05 level, further analysis will be performed to check whether their effects are independent (46).

Statistical analyses related to secondary outcomes 8 and 9 (see pages 4-5) focus on the incremental cost effectiveness ratio (ICER) (47, 48). The ICER relates cost differential to outcome differential between intervention and control groups. Bootstrap methods model statistical uncertainty and the net-benefit approach is applied (49, 50). Maximum willingness to pay thresholds and cost-acceptability curves (51, 52) will be determined. Sensitivity analyses will provide information on robustness of assumptions.

Because no adjustments for multiple endpoints are planned, findings (especially those related to secondary outcomes and subgroup analyses(46)) will be interpreted with caution in view of the number of statistical tests undertaken. Only the result of the primary efficacy analysis will be interpreted in a confirmatory manner. Confirmatory subgroup analyses are not planned. No interim analysis with regard to efficacy will be done.

In case of missing data, multiple imputation will be used to complete the data set for analysis. A complete-case analysis will be performed to assess the influence of missing data and the value of adopting multiple imputation methods.

11.3 Process evaluation

Data collected during NCM-led assessments and delivery of the intervention will be exported by from the ICW Care Manager in pseudonymized format to perform a process evaluation. This will include data related to: participation in DMPs, hospitalizations, use of social services, existence of patient advance directives, clinical parameters (blood pressure, body weight, height, blood sugar, use of podiatrists, use of psychotherapy, participation in diabetes education, results of clinical assessments and actions taken.

11.4 Sample size calculation

Sample size was calculated based on the expected difference between the two treatment groups in mean change in the SDSCA-G score from baseline (T0) compared to 9 months after baseline (T1). Based on data from published studies, which used the revised SDSCA as outcome measure (53-55) we estimated a mean change of 0.5 days (standard deviation 2.0) in the overall SDSCA-G "score" (calculated as the sum of days of items 1 - 10 divided by 10) per patient in 9 months as minimal clinically relevant change.

Based on these estimates, a total of 506 patients (253 per arm) would be required to detect an effect size (Cohen's d) of 0.25 between-groups (PTC vs. TAU) (50% relative increase in self-care) with a power of 80 % applying a two-sided t-test for two independent samples at a significance level of 5%.

Assuming a drop-out rate of 15% over the period of 9 months, the overall required sample size amounts to a total of 582 participants (291 per arm).

12. Concomitant therapy

All participants undergo usual care according to individual needs, which means that the intervention is supplementary to and not replacing usual care.

13. Safety laboratory

Not applicable.

14. Termination criteria

14.1 Individual

PCPs and patients can withdraw their consent at any time without indication of reasons. Data of patients withdrawing their consent during the course of the study will feed into the analysis in form of an ITT analysis if baseline data collection has been completed.

14.2 General

Reasons for general termination of the study are: new insights and findings of other studies, which make the conduct of this study dispensable.

15. Ethics and legal aspects

The study is being conducted in accordance with medical professional codex and the Helsinki Declaration (2008). The study is also in accordance with German Federal Data Security Law (BDSG). All professionals participating in the study oblige to adhere to the abovementioned declarations and laws.

Participation for PCPs, patients and/or their relatives is voluntary. Consents can be withdrawn at any time without any consequences for patients' (usual) care. All patients will be informed about aims, content, duration and process of the trial, particularly as far as risks and unintended consequences are concerned, through written information brochures and through face-to-face communication with their PCP physician.

Data will be collected and analyzed in pseudonymized form for patients, relatives, physicians, and NCMs. Data obtained from DAs will be anonymized.

Pseudonymization will occur as follows: PCPs will be numbered 1-30, the key remains in the intervention coordinating center (GGM). Patients recruited in each PCP will obtain a pseudonym consisting of a combination of the PCP number and a running number (e.g. 3-15 for patient number 15 in PCP number 3). The key remains in the recruiting PCP. Relatives obtain a pseudonym consisting of the letter "R" plus the patient pseudonym (e.g. R-3-15 for the relative of patient number 15 in PCP number 3). No key will be generated for patient's relatives. NCMs will be numbered 1-11. The key remains in the intervention coordinating center (GGM). Access to all keys is restricted for anyone except for the key-holders.

Special emphasis in the information brochure will be paid to the following privacy issue: During the data entry of physician chart-reviews into the ICW Care Manager at T1/T2, the authorized employee of GGM will have access to electronic patient records data, including full names, dates of birth, and assessment results of the preceding time-period since T0 for all patients in the intervention arm. This means that pseudonymization will not be ensured during data entry at T1 and T2.

For the data analysis, however, all data will be exported by the authorized data manager at the study central office (AMV) fully pseudonymized.

This privacy issue will be handled as follows:

(i) through the written informed consent, in which we explicitly mention that patients' identity may be disclosed during data entry at T1/T2, and

(ii) through a written declaration of confidentiality which must be signed by the authorized employee of GGM in order to qualify for the data entry.

Patients' relatives will be informed about aims, content, duration and process of the trial, particularly as far as risks and unintended consequences are concerned, through written information brochures. Written informed consent will be obtained from patients' relatives.

Further privacy issues are related to the hardware- and software-infrastructure, that is deployed in this study in the scope of the ICW Care Manager. The software will be deployed on a secured server of the Center for Information- and Medical Technologies (Zentrum für Informations- und Medizintechnologie, ZIM).

The system-architecture for this project draws upon two virtual servers: an application-server and a database-server (including a test-system). A general proxy server precedes these servers to ensure security of data stored on the database-server. Access to the application-server via internet will be necessary for the NCM to conduct the intervention, and physician reported data entered at T0-T2 by the GGM employee will also be transferred via internet. Both external communication (proxy-server with the internet) and internal communication (proxy-server with the application server) will be encrypted (HTTPS).

Access control to the application is guaranteed via person/entity authorization using a username and password combination that is transmitted over a secure channel (TLS). ICW Care Manager implements TLS 1.1 encryption to guard against unauthorized access to protected health information that is being transmitted over an electronic communications network. Passwords are salted with a 128-bit salt and hashed using a cryptographically strong mechanism. Password complexity is enforced by the application according to the password complexity configured by the customer.

Access control in the application is ensure via user administration, which allows definition of roles and permission to restrict functionality (read, write, create, update, delete) on an GUI level and to restrict access to data objects. Patient level security is guaranteed via restriction of visibility of patients and all patient related data to a user or a group of users (e.g. to guarantee that NCMs only have access to digital records of 'their' patients in the intervention am, but not to those of other NCMs or to the control group).

Audit control includes a GUI based audit feature, which allows a user with the relevant permissions (e.g. the authorized clinical monitor) to query an audit log that contains entries of all relevant accesses on the system with specification of accessing user, date, time, type, scope and accessed data object. Furthermore, through technical, file based logging the application itself logs technical information to disk based log files that may contain relevant information such as system startup, shutdown timestamps and import processes.

The study protocol will be submitted to the ethics committee of the Medical Faculty of the University of Heidelberg prior to the start of the study. Neither patients nor their relatives will be recruited before obtaining the committee's approval.

As mandated by the International Committee of Medical Journal Editors (ICMJE), the trial will be registered in a clinical trial registry after obtaining ethical approval.

16. References

1. Wolff JL, Starfield B, Anderson G. PRevalence, expenditures, and complications of multiple chronic conditions in the elderly. Archives of Internal Medicine. 2002;162(20):2269-76.

2. Sachverständigenrat. Koordination und Integration - Gesundheitsversorgung in einer Gesellschaft des längeren Lebens. . 2009 Sondergutachten 2009.

3. Valderas JM, Starfield B, Sibbald B, Salisbury C, Roland M. Defining Comorbidity: Implications for Understanding Health and Health Services. The Annals of Family Medicine. 2009;7(4):357-63.

4. Wagner EH, Austin BT, Davis C, Hindmarsh M, Schaefer J, Bonomi A. Improving chronic illness care: translating evidence into action. Health Aff (Millwood). 2001;20(6):64-78.

5. Rosenthal TC. The medical home: growing evidence to support a new approach to primary care. Journal of the American Board of Family Medicine : JABFM. 2008;21(5):427-40.

6. Freund T, Peters-Klimm F, Rochon J, Mahler C, Gensichen J, Erler A, et al. Primary care practice-based care management for chronically ill patients (PraCMan): study protocol for a cluster randomized controlled trial [ISRCTN56104508]. Trials. 2011;12(1):163.

7. Bodenheimer TB-M, R. Care management of patients with complex health care needs. Robert Wood Johnson Foundation; 2009.

8. Boult C, Wieland GD. Comprehensive primary care for older patients with multiple chronic conditions: "Nobody rushes you through". JAMA : the journal of the American Medical Association. 2010;304(17):1936-43.

9. Gomersall T, Madill A, Summers LK. A metasynthesis of the self-management of type 2 diabetes. Qual Health Res. 2011;21(6):853-71.

10. Toobert DJ, Hampson SE, Glasgow RE. The summary of diabetes self-care activities measure: results from 7 studies and a revised scale. Diabetes care. 2000;23(7):943-50.

11. Fisher EB, Chan JCN, Nan H, Sartorius N, Oldenburg B. Co-occurrence of diabetes and depression: Conceptual considerations for an emerging global health challenge. Journal of Affective Disorders. 2012;142, Supplement(0):S56-S66.

12. Norris SL, Engelgau MM, Venkat Narayan KM. Effectiveness of Self-Management Training in Type 2 Diabetes: A systematic review of randomized controlled trials. Diabetes Care. 2001;24(3):561-87.

13. Renders CM, Valk GD, Griffin SJ, Wagner EH, Eijk van JT, Assendelft WJJ. Interventions to Improve the Management of Diabetes in Primary Care, Outpatient, and Community Settings: A systematic review. Diabetes Care. 2001;24(10):1821-33.

14. Glazier RH, Bajcar J, Kennie NR, Willson K. A Systematic Review of Interventions to Improve Diabetes Care in Socially Disadvantaged Populations. Diabetes Care. 2006;29(7):1675-88.

15. Pal KE, Sophie V; Michie , Susan; Farmer, Andrew J.; Barnard, Maria L.; Peacock, Richard; Wood,Bindie; Inniss, Joni D.; Murray, Elizabeth Computer-based diabetes self-management interventions for adults with type 2 diabetes mellitus. Cochrane Database of Systematic Reviews. 2013(3).

16. Busse R. Disease management programs in Germany's statutory health insurance system. Health Aff (Millwood). 2004;23(3):56-67.

17. Smith SM, Soubhi H, Fortin M, Hudon C, O'Dowd T. Managing patients with multimorbidity: systematic review of interventions in primary care and community settings. Bmj. 2012;345:e5205.

18. Du SY, C. Evalutation of patient self-management outcomes in health care: a systematic review. Int Nurs Rev. 2010;2:159-67.

19. Gandhi GY, Murad M, Fujiyoshi A, et al. PAtient-important outcomes in registered diabetes trials. JAMA : the journal of the American Medical Association. 2008;299(21):2543-9.

20. Rosemann T, Joos S, Laux G, Gensichen J, Szecsenyi J. Case management of arthritis patients in primary care: a cluster-randomized controlled trial. Arthritis and rheumatism. 2007;57(8):1390-7.

21. Gensichen J, von Korff M, Peitz M, Muth C, Beyer M, Guthlin C, et al. Case management for depression by health care assistants in small primary care practices: a cluster randomized trial. Annals of internal medicine. 2009;151(6):369-78.

22. Peters-Klimm F, Campbell S, Hermann K, Kunz CU, Muller-Tasch T, Szecsenyi J, et al. Case management for patients with chronic systolic heart failure in primary care: the HICMan exploratory randomised controlled trial. Trials. 2010;11:56.

23. Craig P, Dieppe P, Macintyre S, Michie S, Nazareth I, Petticrew M, et al. Developing and evaluating complex interventions: the new Medical Research Council guidance. BMJ. 2008;337:a1655.

24. Eigenmann CA, Colagiuri R, Skinner TC, Trevena L. Are current psychometric tools suitable for measuring outcomes of diabetes education? Diabetic Medicine. 2009;26(4):425-36.

25. Majumdar SJ, JA.; Bowker,SL; Booth,GL.; Dolovich,L.; Ghali,W.; harris,S.;Hux,JE.;Holbrook,A.;Lee HN.;Toth,EL.;Yale,J. A Canadian Consensus for the Standardized Evaluation of Quality Improvement Interventions in Type 2 Diabetes. Canadian Journal of Diabetes. 2005;29(3):220.

26. Freund T, Gensichen J, Goetz K, Szecsenyi J, Mahler C. Evaluating self-efficacy for managing chronic disease: psychometric properties of the six-item Self-Efficacy Scale in Germany. Journal of Evaluation in Clinical Practice. 2013;19(1):39-43.

27. Roick C. KR, Matschinger H., Bernert S., Mory C., Angermeyer MC. Die deutsche Version des Client Sociodemiographic and Service Receipt Inventory. Psychiat Prax. 2001;28(Sonderheft 2):84-90.

28. Chrisholm DK, MR.; Knudsen, HC.; Amaddeo, F.; Gaite, L.; van Wijngaarden, B. Client Socio-Demographic and Service Receipt Inventory--European Version: development of an instrument for international research. EPSILON Study 5. European Psychiatric Services: Inputs Linked to Outcome Domains and Needs. . Br J Psychiatry. 2000;Suppl(39):28.

29. Levac D, Colquhoun H, O'Brien K. Scoping studies: advancing the methodology. Implementation Science. 2010;5(1):69.

30. Kerr EA, Heisler M, Krein SL, Kabeto M, Langa KM, Weir D, et al. Beyond comorbidity counts: how do comorbidity type and severity influence diabetes patients' treatment priorities and self-management? Journal of general internal medicine. 2007;22(12):1635-40.

31. Calvert M, Blazeby J, Altman DG, Revicki DA, Moher D, Brundage MD, et al. Reporting of patient-reported outcomes in randomized trials: the CONSORT PRO extension. JAMA : the journal of the American Medical Association. 2013;309(8):814-22.

32. Goetz K, Freund T, Gensichen J, Miksch A, Szecsenyi J, Steinhaeuser J. Adaptation and psychometric properties of the PACIC short form. The American journal of managed care. 2012;18(2):e55-60.

33. ÄZQ. PAID – Problembereiche bei Diabetes (Kurzform). 1.0 ed: BÄK; KBV; AWMF; 2012.

34. Psychiatric Research Unit WCCfMH, Frederiksborg General Hospital. WHO (Fünf) - FRAGEBOGEN ZUM WOHLBEFINDEN. 1998 ed: Psychiatric Research Unit, WHO Collaborating Center for Mental Health, Frederiksborg General Hospital; 1998.

35. Rabin R, de Charro F. EQ-5D: a measure of health status from the EuroQol Group. Annals of medicine. 2001;33(5):337-43.

36. Baden-Württemberg S. AUDIT-C-Fragebogen. UKL Freiburg.

37. Schenk L, Bau AM, Borde T, Butler J, Lampert T, Neuhauser H, et al. [A basic set of indicators for mapping migrant status. Recommendations for epidemiological practice]. Bundesgesundheitsblatt, Gesundheitsforschung, Gesundheitsschutz. 2006;49(9):853-60.

38. Bundesamt S. Demographische Standards: Ausgabe 2010. Wiesbaden: Statistisches Bundesamt; 2010.

39. Noll HW, S. Schichtzugehörigkeit nicht nur vom Einkommen bestimmt. Informationsdienst Soziale Indikatoren. 2011;45:1-6.

40. IQWIG. Abschlussbericht: Urin- und Blutzuckerselbstmessung bei Diabetes mellitus Typ 2. Instut für Qualität und Wirtschaftlichkeit im Gesundheitswesen, 2009 14.10.2009. Report No.: A05-08, Version 1.0.

41. Malanda UL WL, Riphagen II, Dekker JM, Nijpels G, Bot SDM. Self-monitoring of blood glucose in patients with type 2 diabetes mellitus who are not using insulin. Cochrane Database of Systematic Reviews. 2012(1).

42. Farmer AJ, Perera R, Ward A, Heneghan C, Oke J, Barnett AH, et al. Meta-analysis of individual patient data in randomised trials of self monitoring of blood glucose in people with non-insulin treated type 2 diabetes. Bmj. 2012;344.

43. Richtlinie des gemeinsamen Bundesausschusses zur Umsetzung der Regelungen in § 62 für schwerwiegende chronische Erkrankte ("Chroniker Richtlinie"). Sect. §2 (2008).

44. Perera R, Heneghan C, Yudkin P. Graphical method for depicting randomised trials of complex interventions. BMJ. 2007;334(7585):127-9.

45. Tugwell P, de Savigny D, Hawker G, Robinson V. Equity-Effectiveness Loop: Working against the odds: The application of clinical epidemiologic methods to health equity. BMJ. 2005.

46. Rothwell PM. Subgroup analysis in randomised controlled trials: importance, indications, and interpretation. The Lancet.365(9454):176-86.

47. Salize HJ KR. Gesundheitsökonomie in der Psychiatrie: Konzepte, Methoden, Analysen. 1 ed. Stuttgart: Kohlhammer; 2010.

48. Glick HD, JA; Sonnad, SS; Polsky, D. Economic evaluation in clinical trials. Oxford: Oxford University Press; 2007.

49. Carpenter J BJ. Bootstrap confidence intervals: when, which, what? A practical guide for medical statisticians. Stat Med. 2000;19(9):1141–64.

50. Löthgren M ZN. Definition, interpretation and calculation of cost-effectiveness acceptability curves. . Health Econ 2000;9(7):623–30.

51. Fenwick E MD, Levy AR, Nichol G. Using and interpreting cost-effectiveness acceptability curves: an example using data from a trial of management strategies for atrial fibrillation. BMC Health Serv Res 2006;6(52).

52. Hoch JS BA, Willan AR. Something old, something new, something borrowed, something blue: a framework for the marriage of health econometrics and cost-effectiveness analysis. . Health Econ 2002;11(5):415-30.

53. Tang TS, Funnell MM, Noorulla S, Oh M, Brown MB. Sustaining short-term improvements over the long-term: Results from a 2-year diabetes self-management support (DSMS) intervention. Diabetes Research and Clinical Practice. 2012;95(1):85-92.

54. Wangberg SC. An Internet-based diabetes self-care intervention tailored to self-efficacy. Health Education Research. 2008;23(1):170-9.

55. Lee H, Ahn S, Kim Y. Self-care, Self-efficacy, and Glycemic Control of Koreans With Diabetes Mellitus. Asian Nursing Research. 2009;3(3):139-46.

17. Unterschriften

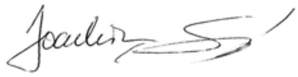

Prof. Dr. med. Dipl. Soz. Joachim Szecsenyi,

Ärztlicher Direktor, FA für Allgemeinmedizin

18. Appendices

- Hintergrundinformation für Patienten (Version 1, 22.10.2013)
- Hintergrundinformation für Angehörige (Version 1, 22.10.2013)
- Hintergrundinformation für Ärzte (Version 1, 22.10.2013)
- Hintergrundinformation für MFA/Fallmanager (Version 1, 22.10.2013)
- Hintergrundinformation für Dialogassistenten (Version 1, 22.10.2013)
- Teilnahme- und Einverständniserklärung für Patienten (Version 1, 22.10.2013)
- Teilnahme- und Einverständniserklärung für Angehörige (Version 1, 22.10.2013)
- Teilnahme- und Einverständniserklärung für Ärzte (Version 1, 22.10.2013)
- Teilnahme- und Einverständniserklärung für MFA/Fallmanager (Version 1, 22.10.2013)
- Teilnahme- und Einverständniserklärung für Dialogassistenten(Version 1, 22.10.2013)
- CV Studienleitung
- Patientenfragebogen T0-T2 (Version 1, 29.10.2013)
- Angehörigenfragebogen zur Erfassung des „Financial Family burden“ (Version 1, 07.10.2013)
- Arztfragebogen für Ersterhebung T0 (Ausdruck der digitalen Version, Abweichungen von der Papierform sind gelb markiert und werden in der Papierform NICHT erhoben) (Version 1.1, 06.08.2013)
- Arztfragebogen für Enderhebung T1 (Ausdruck der digitalen Version, Abweichungen von der Papierform sind gelb markiert und werden in der Papierform NICHT erhoben) (Version 1.1, 06.08.2013)

- Arztfragebogen für Follow-up-Erhebung T2 (Ausdruck der digitalen Version, Abweichungen von der Papierform sind gelb markiert und werden in der Papierform NICHT erhoben) (Version 1.1, 06.08.2013)
- Eingangsfragebogen Ärzte (Version 1, 22.10.2013)
- Eingangsfragebogen MFA (Version 1, 22.10.2013)
- Eingangsfragebogen DA (Version 1, 22.10.2013)
- Studienhilfsmittel:
    - Checkliste Telefonmonitoring (digitale Checkliste, Version 1.1, 06.08.2013)
    - Formular „Erstkontakt Assessment“ (digitale Checkliste, Version 1.1, 06.08.2013)
    - Formular „Folgekontakt Assessment“ (digitale Checkliste, Version 1.1, 06.08.2013)
    - Meldebogen für unerwünschte Ereignisse (Version 1, 22.10.2013)
    - Verschwiegenheitserklärung Datenmanager GGM (Version 1, 19.09.2013)
